# The AP-1 transcription factor JunB is required for Th17 cell differentiation

**DOI:** 10.1038/s41598-017-17597-3

**Published:** 2017-12-12

**Authors:** Soh Yamazaki, Yoshihiko Tanaka, Hiromitsu Araki, Akira Kohda, Fumiyuki Sanematsu, Tomoko Arasaki, Xuefeng Duan, Fumihito Miura, Takaharu Katagiri, Ryodai Shindo, Hiroyasu Nakano, Takashi Ito, Yoshinori Fukui, Shogo Endo, Hideki Sumimoto

**Affiliations:** 1 Department of Biochemistry, Kyushu University Graduate School of Medical Sciences, Fukuoka, 812-8582 Japan; 2 Division of Immunogenetics, Department of Immunobiology of Neuroscience, Medical Institute of Bioregulation, Kyushu University, Fukuoka, 812-8582 Japan; 3 Section of Infection Biology, Department of Functional Bioscience, Fukuoka Dental College, Fukuoka, 814-0193 Japan; 4 Aging Neuroscience Research Team, Tokyo Metropolitan Institute of Gerontology, Tokyo, 173-0015 Japan; 5 Department of Biochemistry, Toho University School of Medicine, Tokyo, 143-8540 Japan

## Abstract

Interleukin (IL)-17-producing T helper (Th17) cells are crucial for host defense against extracellular microbes and pathogenesis of autoimmune diseases. Here we show that the AP-1 transcription factor JunB is required for Th17 cell development. *Junb*-deficient CD4^+^ T cells are able to develop *in vitro* into various helper T subsets except Th17. The RNA-seq transcriptome analysis reveals that JunB is crucial for the Th17-specific gene expression program. *Junb*-deficient mice are completely resistant to experimental autoimmune encephalomyelitis, a Th17-mediated inflammatory disease, and naive T helper cells from such mice fail to differentiate into Th17 cells. JunB appears to activate Th17 signature genes by forming a heterodimer with BATF, another AP-1 factor essential for Th17 differentiation. The mechanism whereby JunB controls Th17 cell development likely involves activation of the genes for the Th17 lineage-specifying orphan receptors RORγt and RORα and reduced expression of Foxp3, a transcription factor known to antagonize RORγt function.

## Introduction

Interleukin (IL)-17–producing CD4^+^ T helper (Th17) cells are a key immune cell lineage that protects against extracellular microbe infection^1,2^ and is critically associated with inflammatory autoimmune diseases, e.g., experimental autoimmune encephalomyelitis (EAE), a mouse model for multiple sclerosis^3,4^. Commitment of naive CD4^+^ T cells to the Th17 lineage occurs when T cells are stimulated in the presence of IL-6 and transforming growth factor-β (TGF-β). Th17 cells are characterized by expression of the cytokines IL-17A, IL-17F, and IL-21, and the IL-23 receptor (IL-23R). These molecules are required for effector functions of Th17 cells and also crucial for stabilization of the Th17 phenotype and acquisition of pathogenicity.

Differentiation from naive CD4^+^ T cells into Th17 cells is driven by the lineage-specifying orphan nuclear receptors RORγt (encoded by *Rorc*)^5^ and RORα (encoded by *Rora*)^6^. To achieve the full Th17 differentiation program, however, networks of multiple transcription factors are required. For instance, Th17 cell development requires rapidly-induced transcription factors such as basic leucine zipper transcription factor, ATF-like (BATF)^7^ and interferon regulatory factor 4 (IRF4)^8^, both of which positively regulate expression of ROR proteins and thus control Th17 cell specification^7,8^. RORγt expression is also elevated by the transcription factor STAT3, which functions downstream of the receptor for the Th17-polarizing cytokine IL-6 and plays a crucial role in Th17 cell differentiation^9^. By contrast, IκBζ, a nuclear protein that acts with NF-κB as a transcriptional activator in immune cells^10–12^, has been reported to promote development of Th17 cells without affecting expression of *Rorc* and *Rora* ^13^. On the other hand, Foxp3, a transcription factor that specifies the lineage of regulatory T (Treg) cells, prevents Th17 cell differentiation by antagonizing RORγt function^14^.

BATF is known to function not only in Th17 cells but also in a variety of immune cells, which is probably mediated by leucine-zipper-mediated formation of the activator protein-1 (AP-1) complex with a transcription factor of the Jun family^15–18^. This family contains three members, i.e., c-Jun, JunB, and JunD. In spite of their amino acid sequence similarity, the Jun family AP-1 proteins have distinct functions; e.g., whereas mice lacking JunD are viable, c-Jun-deficient embryos die at embryonic day 12.5 (E12.5) with abnormalities in liver and heart, and JunB inactivation causes multiple defects in extra-embryonic tissues, leading to embryonic lethality at E8.5–10.5^19^. Although the three Jun proteins are each capable of effectively binding to the Th17-polarizing AP-1 factor BATF^15–18^, their role in Th17 development has remained to be elucidated.

In the present study, we show that JunB is required for Th17 cell differentiation. CD4^+^ T cells deficient in the JunB-encoding gene *Junb* fail to differentiate into Th17 cells. The critical role of JunB in generation of the Th17-specific gene expression pattern is presented by the RNA-seq transcriptome analysis. *Junb*-deficient mice are completely resistant to EAE. The dominant role of JunB as a partner of BATF is consistent with the findings that c-Jun is much less abundantly expressed in Th17 cells compared with JunB and JunD, and that JunB but not JunD cooperates with BATF to activate Th17 signature genes. JunB appears to control Th17 cell specification by inducing activation of *Rorc* and *Rora* and by reducing expression of Foxp3.

## Results

### JunB-deficient T helper cells fail to differentiate into Th17 cells

**Figure 1:**
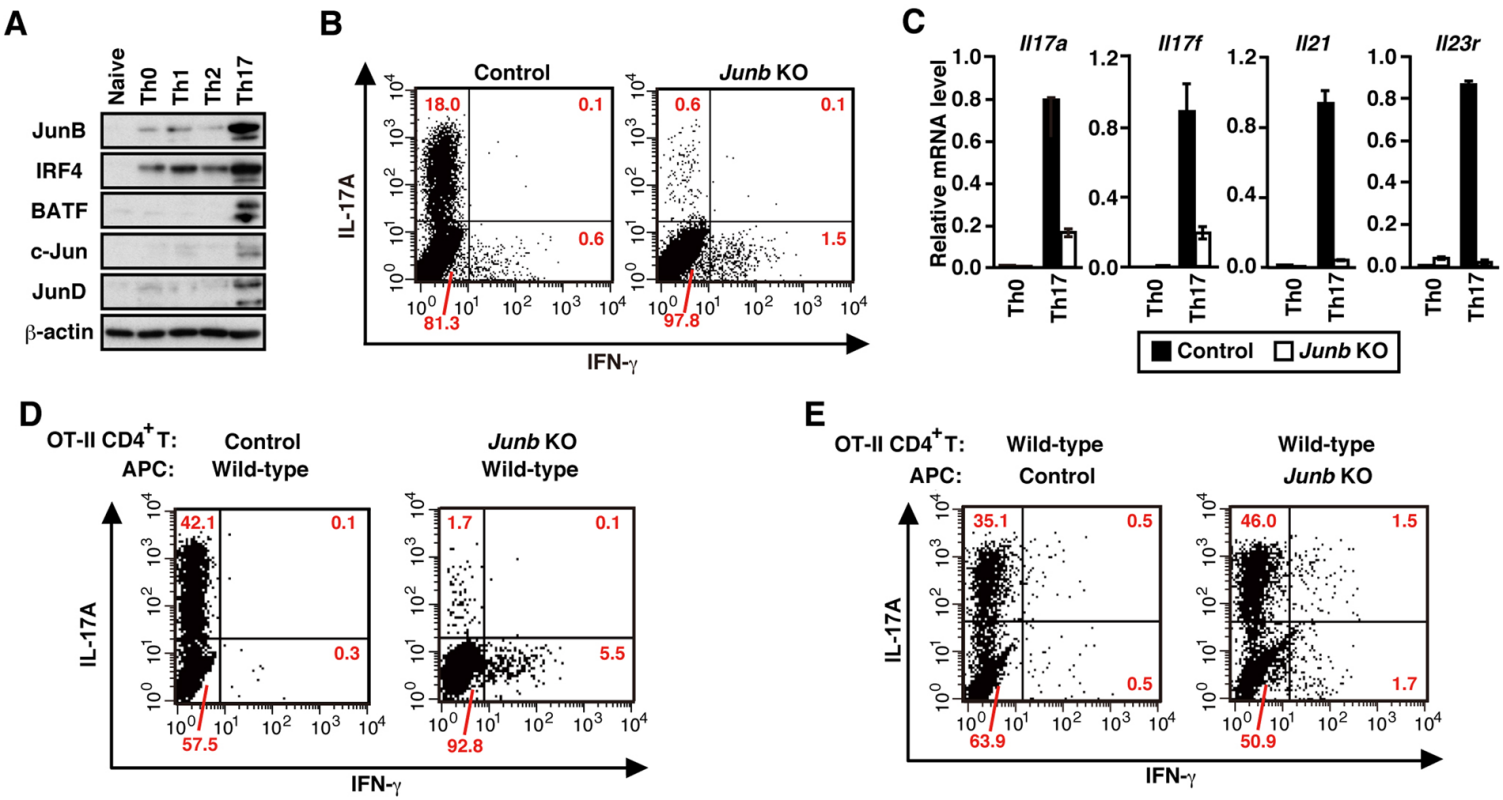
JunB is required for Th17 differentiation *in vitro*. (**A**) Immunoblot analysis of expression of Jun family proteins in wild-type CD4^+^ T cells differentiated *in vitro* under the indicated conditions. (**B**) Flow cytometric analysis of IL-17A production in CD4^+^ T cells cultured under Th17-polarizing conditions. (**C**) Real-time PCR analysis of expression of Th17 signature genes in CD4^+^ T cells cultured under the indicated conditions. Data are presented as mean ± SD. (**D** and **E**) IL-17A production in *Junb*-deficient (KO) OT-II CD4^+^ T cells co-cultured with control (**D**) or *Junb*-deficient (**E**) APCs in the presence of OVA_323–339_. *Junb* KO, *Meox2* ^Cre^; *Junb* ^f/f^ mice; Control, littermate mice with *Junb* ^+/+^ (**A**,**B**,**D**, and **E**) or *Junb* ^f/+^ (**C**).

To know the mechanism underlying Th17 cell differentiation, we immunoprecipitated the Th17-polarizing transcription factor IκBζ and analyzed IκBζ-interacting proteins by liquid chromatography-tandem mass spectrometry (LC-MS/MS) and by various binding assays (Supplementary Figure 1). The analyses led to identification of JunB as a novel IκBζ-binding protein, raising a possibility that JunB also participates in Th17 development. Indeed JunB expression was markedly induced, when naive CD4^+^ T cells were activated via T cell receptor under Th17 cell-polarizing conditions (IL-6 and TGF-β) (Fig. 1A). To investigate the role of JunB in Th17 cell differentiation, we generated *Junb* ^f/f^ mice (Supplementary Figure 2A–F); the mice were crossed to *Meox2* ^+/Cre^ mice for deletion of the *Junb* locus in the embryo proper but not in extraembryonic tissues, because conventional *Junb*-deficient mice are known to be embryonic lethal due to placental defects^20^.

Consistent with previous reports using similar *Junb*-deficient mice^20,21^, the present *Junb*-deficient mice also exhibited myeloproliferative abnormality (Supplementary Figure 3A) and impairment of osteoclast differentiation (Supplementary Figure 3B,C). Ablation of *Junb* did not affect development of naive CD4^+^ T cells (Supplementary Figure 3D,E). On the other hand, when *Junb*-deficient CD4^+^ T cells cultured under Th17-polarizing conditions, they expressed much less amounts of IL-17A (Fig. 1B) and *Il17a* mRNA (Fig. 1C) than control CD4^+^ T cells. Furthermore, expression of other Th17 signature genes encoding IL-17F (*Il17f*), IL-21 (*Il21*), and IL-23R (*Il23r*) was diminished in *Junb*-deficient T cells (Fig. 1C), indicating an essential role for JunB in Th17 cell differentiation. When OT-II mice-derived CD4^+^ T cells^22^ were cultured with the chicken ovalbumin peptide OVA_323–339_ and splenic antigen-presenting cells (APCs) under Th17-polarizing conditions, *Junb-*deficient OT-II T cells differentiated into IL-17A-producing cells much less efficiently than control cells (Fig. 1D). JunB appears to function in T cells but not in APCs, because differentiation of control naive T cells occurred even in the presence of *Junb-*deficient APCs (Fig. 1E). Thus, JunB likely plays a crucial role in Th17 cell differentiation.

### JunB is crucial for Th17-specific gene expression program

**Figure 2:**
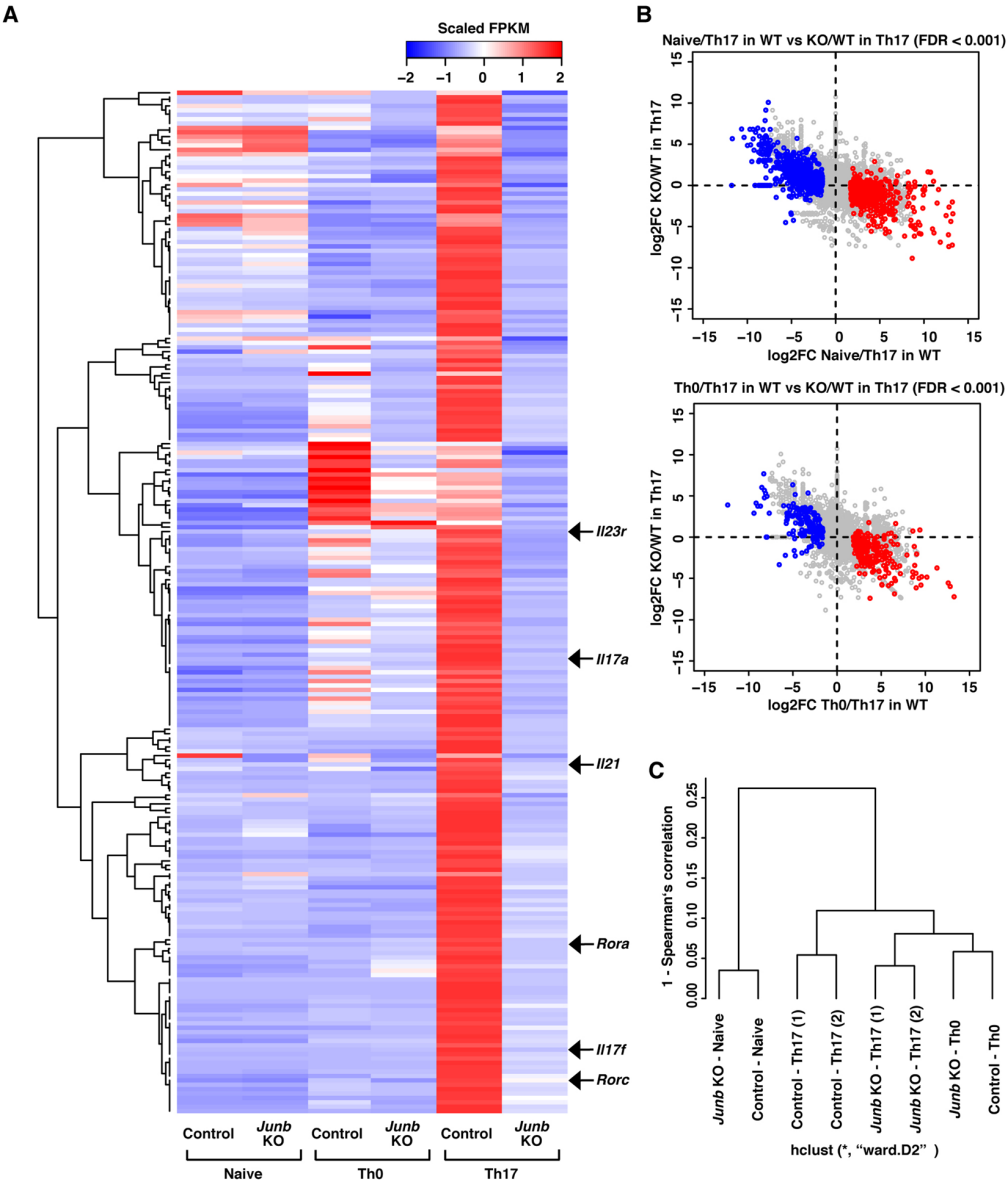
JunB governs the Th17-specific gene expression program. (**A**) Heat map of hierarchical clustering of 234 genes down-regulated in *Junb*-deficient Th17 cells compared to control Th17 cells. Each column displays scaled FPKM of 234 genes in the indicated sample. Arrows indicate typical Th17 signature genes. (**B**) Correlation between JunB-regulated genes and those differentially expressed during Th17 differentiation. For each gene, log2FC between control and *Junb*-deficient Th17 cells are plotted against log2FC during Th17 differentiation from control naive or Th0 cells. Red and blue dots indicate genes significantly up- and down-regulated during Th17 differentiation, respectively (FDR < 0.001, |log2FC| > 1). (**C**) Dendrogram of unsupervised hierarchical clustering of the transcriptomes of control and *Junb*-deficient naive, Th0, and Th17 cells.

To investigate the effect of JunB deficiency on global gene expression under Th17-polarizing conditions, we performed RNA-seq analyses. Consistent with the results obtained in the qPCR analyses (Fig. 1C), expression of the Th17 signature genes (*Il17a*, *Il17f*, *Il21*, and *Il23r*) was abrogated in the absence of JunB (Fig. 2A). Pathway enrichment analyses revealed that genes involved in Th17-related functions were significantly enriched in the JunB-regulated gene set (Supplementary Figure 4). A strong correlation was observed between genes up-regulated during Th17 differentiation and those impaired by JunB deficiency (Fig. 2A, red dots): genes highly activated during Th17 differentiation had a tendency to be effectively impaired by the absence of JunB. We also found that expression of another subset of genes was down-regulated during Th17 differentiation in a JunB-dependent manner (Fig. 2B, blue dots): genes severely down-regulated during Th17 differentiation tended to be activated by JunB deficiency (Fig. 2B). The hierarchical clustering analysis revealed that lack of JunB drastically altered gene expression in CD4^+^ T cells after culture under Th17-polarizing conditions (Fig. 2C). By contrast, the absence of JunB did not largely affect expression patterns in naive T helper cells and Th0 cells (Fig. 2C). Importantly, the expression profile of *Junb*-deficient CD4^+^ T cells under Th17-polarizing conditions was more similar to that of normal Th0 cells than that of Th17 cells (Fig. 2C). Thus JunB is indispensable for generation of a gene expression pattern specific to Th17 cells.

### JunB is dispensable for development of Th1, Th2, and Treg cells

**Figure 3:**
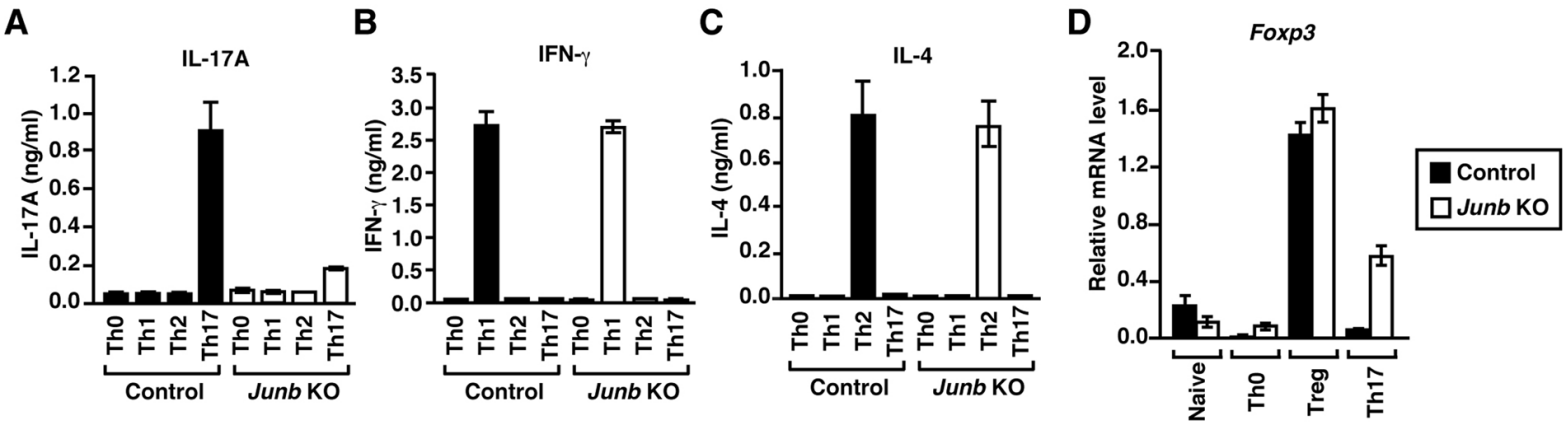
JunB specifically controls Th17 differentiation. (**A**) ELISA analysis of cytokine release from CD4^+^ T cells cultured under the indicated conditions. (**B**) *Foxp3* expression in CD4^+^ T cells cultured under the indicated conditions. *Junb* KO, *Meox2* ^Cre^; *Junb* ^f/f^ mice; Control, littermate mice with *Junb* ^*+/+*^ (**A**) or *Junb* ^f/+^ (**B**). Data are presented as mean ± SD.

In contrast to the essential role of JunB in Th17 differentiation, neither Th1 nor Th2 differentiation was affected by ablation of *Junb*: interferon-γ (IFN-γ) and IL-4 were normally synthesized under Th1- and Th2-polarizing conditions, respectively (Fig. 3A–C). In addition, *Foxp3* (encoding Foxp3), which specifies differentiation into Treg cells^1,2^, was expressed in *Junb-*deficient cells as much as in control cells under Treg-polarizing conditions (Fig. 3D). Intriguingly, *Foxp3* expression under Th17-polarizing conditions was increased in *Junb-*deficient cells (Fig. 3D); a similar increase has been also observed by ablation of *Irf4* and *Batf*, each being required for development of Th17 cells^7,8^. These findings indicate that JunB is selectively required for Th17 differentiation.

### JunB-deficient mice are completely resistant to EAE induction

**Figure 4:**
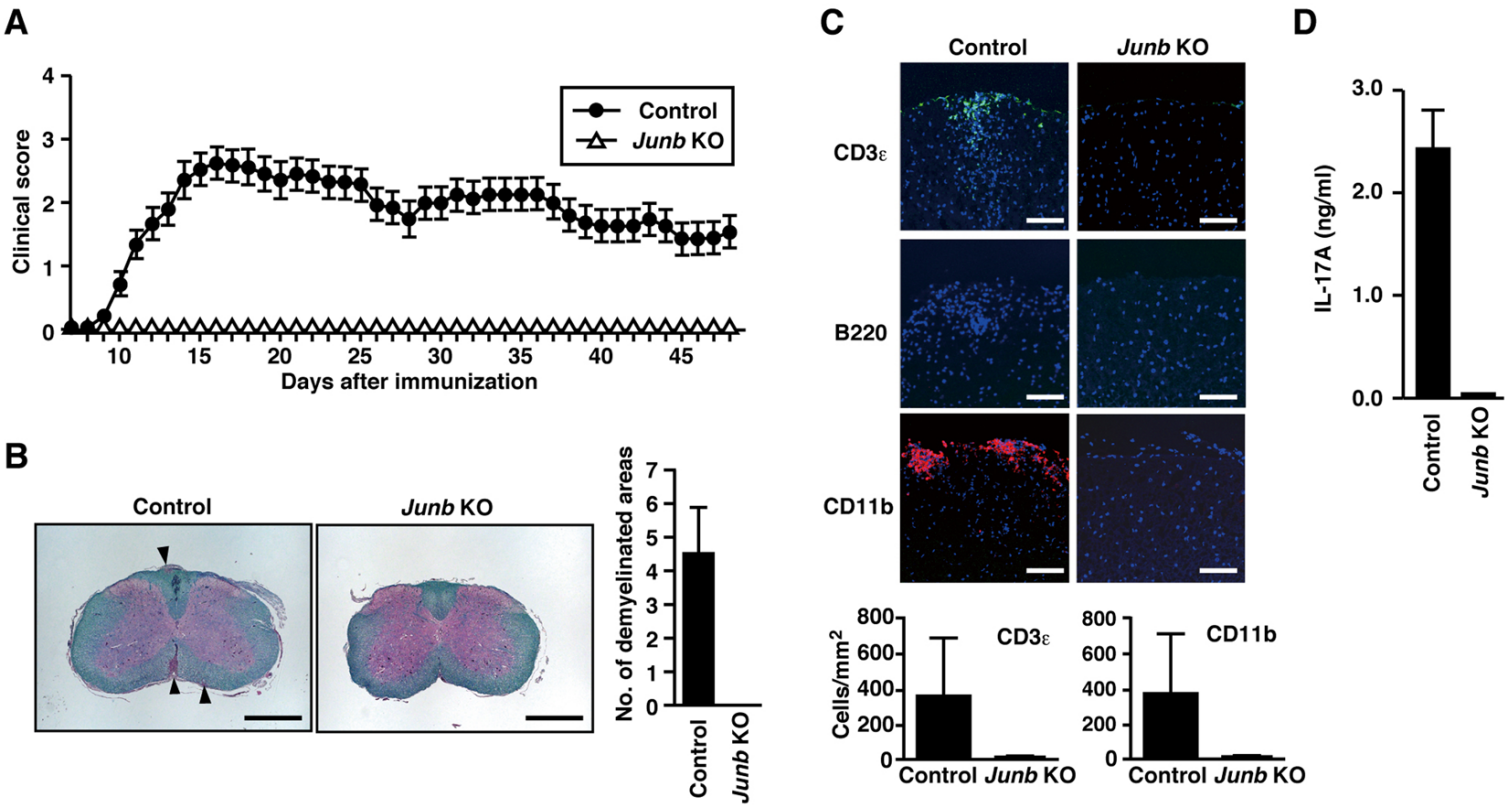
*Junb*-deficient mice are resistant to EAE. (**A**) EAE disease course in *Junb* ^f/+^ (control) (*n* = 25) and *Junb*-deficient (KO) (*n* = 22) mice. Mean clinical scores are presented as mean ± SEM. (**B** and **C**) Pathological analysis in the spinal cord sections of mice on day 12 after EAE induction (*n* = 6). Arrowheads indicate demyelinated areas (Luxol fast blue) in (**B**). Scale bars in (**B**), 500 µm. Infiltrated cells were detected by immunostaining for CD3ε (T cells), B220 (B cells), and CD11b (myeloid cells) in (**C**). Scale bars in (**C**), 100 µm. (**D**) IL-17A production in MOG_35–55_-restimulated CD4^+^ T cells from mice with EAE. Error bars in (**B**,**C** and **D**) represent SD.

To examine the *in vivo* role of JunB in Th17 cell differentiation, we evaluated the effects of *Junb* ablation in EAE, because Th17 cells are the major pathogenic population in this disease^3,4^. *Junb* ^f/+^ mice were immunized with myelin oligodendrocyte glycoprotein peptide 35–55 (MOG_35–55_) and monitored for clinical signs of EAE. As shown in Fig. 4A, all *Junb* ^f/+^ mice (n = 25) developed severe EAE. In contrast, none of the *Junb-*deficient mice (n = 22) displayed signs of paralysis during the 48-day period (Fig. 4A). The EAE phenotype of *Junb*-deficient mice is similar to that of mice lacking *Batf* ^7^ or *Irf4* ^8^. The resistance of *Junb*-deficient mice to EAE induction was confirmed by histopathological analysis of the spinal cords of *Junb* ^f/+^ and *Junb*-deficient mice. As shown in Fig. 4B, three to six demyelinated areas were observed in the spinal cord sections of *Junb* ^f/+^ mice; on the other hand, no demyelinated areas in those of *Junb-*deficient mice (Fig. 4B). In addition, although both CD3ε^+^ T cells and CD11b^+^ myeloid cells were densely infiltrated into the spinal cord of *Junb* ^f/+^ mice, no infiltration of immune cells occurred in *Junb-*deficient mice (Fig. 4C). Thus, clinical and histopathological analyses indicate that *Junb-*deficient mice are completely resistant to EAE.

To investigate the presence or absence of Th17 cells in MOG-treated *Junb*-deficient mice, we prepared CD4^+^ T cells and restimulated them with MOG_35–55_ in the presence of APCs. As shown in Fig. 4D, IL-17A was abundantly expressed upon restimulation in CD4^+^ T cells from MOG-immunized *Junb* ^f/+^ mice. By contrast, CD4^+^ T cells from immunized *Junb-*deficient mice failed to produce IL-17A when stimulated with MOG_35–55_ (Fig. 4D), confirming that Th17 cells are absent from *Junb-*deficient mice. Taken together with the present findings, we conclude that JunB is required for Th17 differentiation both *in vitro* and *in vivo*.

Because epidermis-specific deletion of *Junb* is known to result in skin inflammation^19^, we studied the effect of systemic *Junb* deletion in imiquimod-induced dermatitis, a mouse model for psoriasis-like inflammatory disease^23^. Treatment with imiquimod induced ear swelling in *Junb*-deficient mice to the extent similar to that in control mice (Supplementary Figure 5A). In addition, *Junb* deletion did not affect the induction of psoriasis-associated genes such as *Defb4*, *Il17f*, *S100a9*, and *Il19* in imiquimod-treated skin lesions, although the mRNA level of the two other associated genes *IIl23a* and *Il24* in *Junb*-deficient mice was slightly higher than that in control mice at day 5 after imiquimod treatment (Supplementary Figure 5B). These findings suggest that JunB plays a marginal, if any, role in imiquimod-induced psoriasis.

### c-Jun is much less expressed than JunB and JunD in Th17 cells

**Figure 5:**
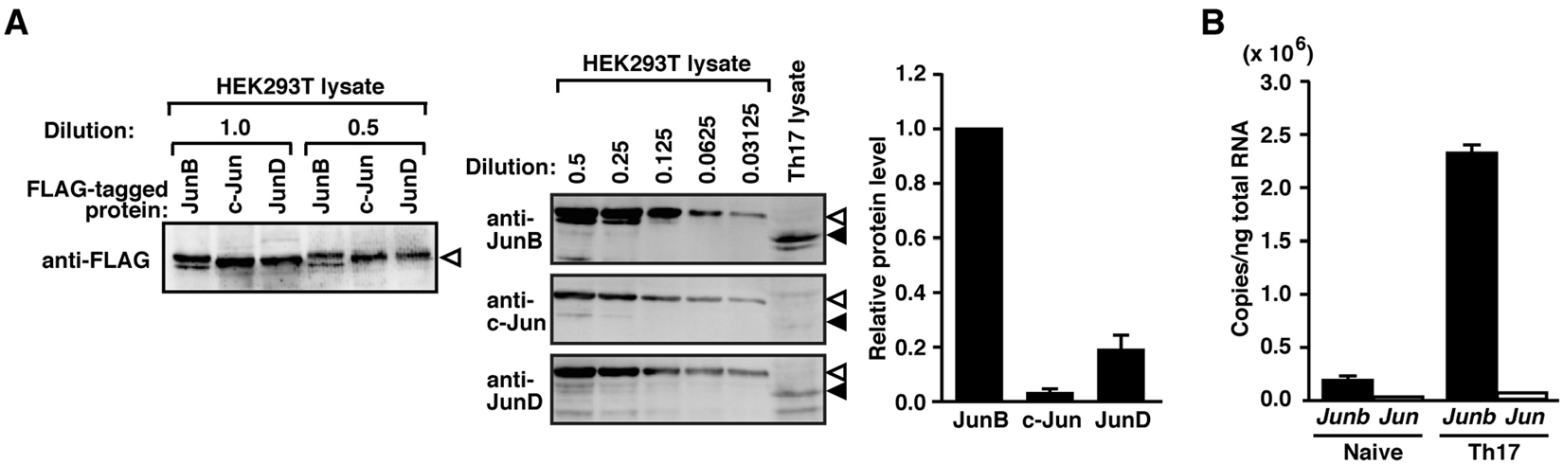
JunB but not c-Jun is abundantly expressed in Th17 cells. (**A**) Immunoblot analysis for evaluation of relative expression levels of endogenous Jun family proteins in Th17 cells. The same amounts of FLAG–JunB, FLAG–c-Jun or FLAG–JunD, which were expressed in HEK293T cells, were estimated by immunoblot with an anti-FLAG antibody (M2) (left panel). Serially diluted proteins and the Th17 cell lysate were subjected to immunoblot analysis with anti-JunB, anti-c-Jun, or anti-JunD antibodies (middle panel), followed by quantification with Odyssey Infrared Imaging System. FLAG-tagged and endogenous proteins were denoted by white and black arrowheads, respectively. Relative protein levels of endogenous JunB, c-Jun, and JunD were shown in the right panel in **(A)**. (**B**) Real-time PCR analysis for relative mRNA copy numbers of *Junb* and *Jun* in naive CD4^+^ T cells and Th17 cells. mRNA copy numbers were estimated from standard curves that were generated using known numbers of a plasmid encoding *Junb* or *Jun*. Data are presented as mean ± SD.

The present finding that single gene ablation of *Junb* is sufficient for effective suppression of Th17 development raised the question why *Junb* plays such an indispensable role in spite of the presence of other Jun family genes. Indeed the two closely-related proteins c-Jun and JunD as well as JunB were each capable of directly interacting with BATF (Supplementary Figure 6A,B), an AP-1 protein that is required for Th17 differentiation^7^, and can exist in a complex with BATF on an AP-1 site, as demonstrated by recent analysis using electrophoretic mobility shift assays (EMSAs)^24–26^. To know the reason for the dominant role of JunB in Th17 development, we first evaluated the relative amounts of the Jun family proteins expressed in Th17 cells. For this purpose, immunoblot analysis was performed for detection of endogenous JunB, c-Jun, and JunD in Th17 cells using the same amounts of the respective FLAG-tagged Jun proteins to make standard curves (see “Methods”; Fig. 5A; Supplementary Figure 7). As estimated by the analysis, c-Jun was much less expressed than JunB in Th17 cells, whereas the amount of JunD protein was slightly smaller than that of JunB (Fig. 5A). Consistent with this, only a marginal expression of mRNA for c-Jun was observed in Th17 cells compared with *Junb* mRNA expression (Fig. 5B). The low expression of c-Jun in Th17 cells appears to agree with the previous observation that c-Jun is not involved in the AP-1 complex in Th17 cells, in contrast to JunB and JunD^25^. In addition, Th17 development was not impaired by knockdown of c-Jun using siRNAs, especially c-Jun siRNA #2, and also c-Jun siRNA #3, but to a lesser extent (Supplementary Figure 8). Thus c-Jun does not appear to play a major role in Th17 development because of its low expression, although c-Jun has an ability to form an AP-1 complex with BATF when overexpressed in HEK293T cells^26^.

### JunB but not JunD cooperates with BATF to activate Th17 signature genes

**Figure 6:**
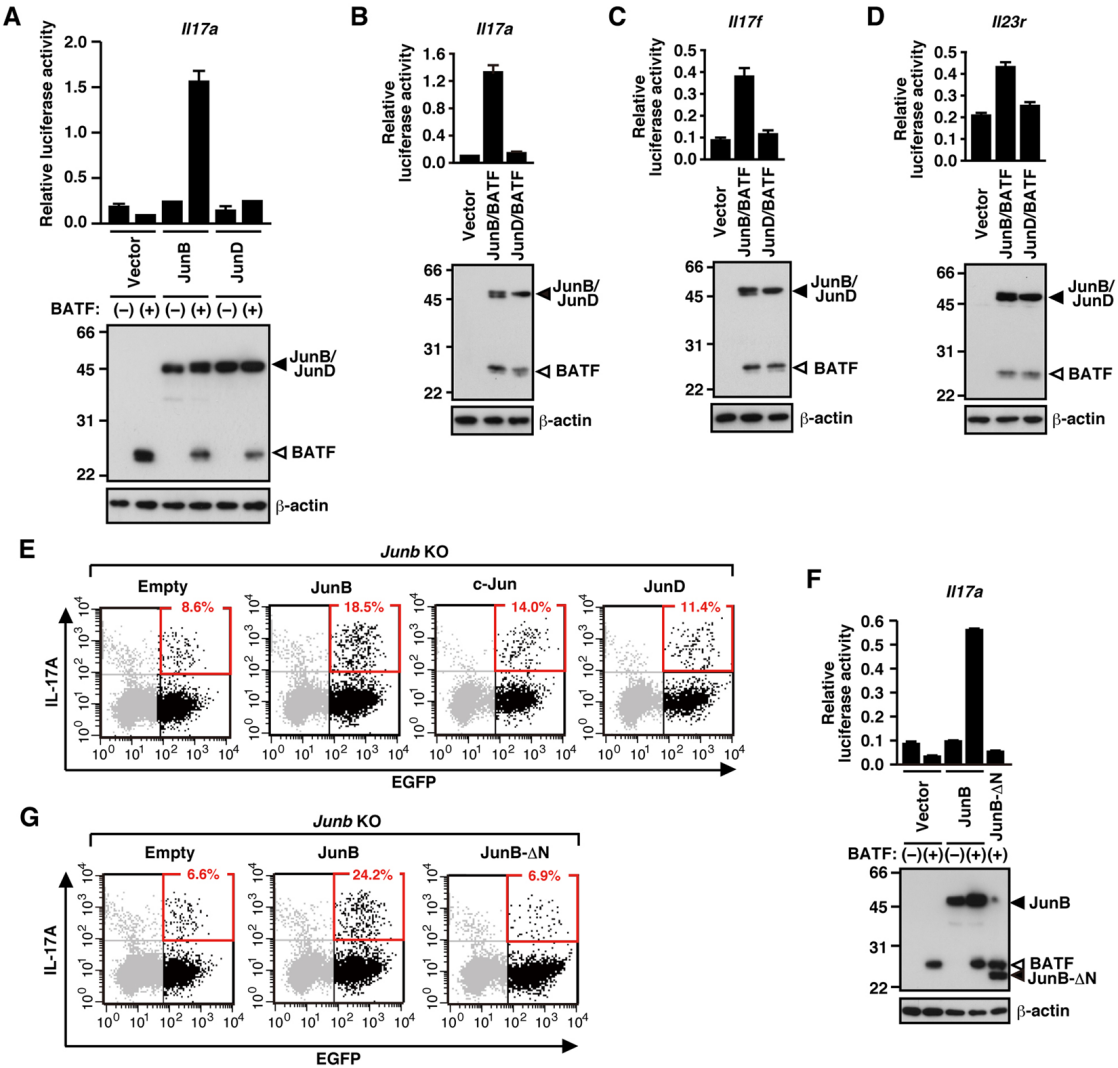
JunB but not JunD regulates expression of Th17 signature genes. (**A**,**B**,**C**,**D** and **F**) Luciferase reporter assay for transcriptional activation of the promoter of *Il17a* (**A**,**B** and **F**), *Il17f* (**C**), or *Il23r* (**D**) in HEK293T (**A** and **F**), NIH3T3 (**B** and **C**), or BW5147α^−^β^−^ (**D**) cells by exogenous expression of Jun family proteins and/or BATF, and immunoblot (IB) analysis of the exogenously expressed proteins. Data are presented as mean ± SD. (**E** and **G**) IL-17A production in *Junb*-deficient (KO) OT-II CD4^+^ T cells by retroviral overexpression of Jun family proteins (**E**) or a N-terminally truncated JunB (JunB-∆N) (**G**) together with EGFP. Production of IL-17A was analyzed by flow cytometry; numbers indicate percent EGFP^+^ cells producing IL-17A.

To further know the reason why JunB plays the dominant role in Th17 development, we next investigated the role for JunB in BATF-dependent activation of Th17 signature genes and compared it with that for JunD, which is present in Th17 cells at the level comparable to JunB in contrast to the low expression of c-Jun. As described above, JunB as well as JunD is capable of forming a heterodimer with BATF. Although JunB by itself failed to induce transcription via the *Il17a* promoter, JunB activated the promoter in cooperation with BATF (Fig. 6A,B), indicating that JunB forms a productive dimer with BATF in *Il17a* activation. On the other hand, JunD did not elicit *Il17a* transcription even in the presence of BATF. Furthermore, JunB but not JunD cooperated with BATF to activate the Th17 signature genes *Il17f* and *Il23r* (Fig. 6C,D). These findings indicate that JunB functions as an indispensable partner of BATF in Th17 development, whereas JunD does not play a major role in regulation of Th17 cells.

We also used *Junb-*deficient CD4^+^ T cells to know the role of Jun family proteins in Th17 differentiation. Compared with JunB, retrovirally expressed JunD only marginally restored Th17 development of the *Junb-*deficient cells (Fig. 6E). The finding supports the idea that JunD is not a major regulator in Th17 differentiation. The activity of JunB likely depends on the N-terminal region, which is not involved in binding to DNA or dimerization with BATF^15^, because truncation of this region resulted in a loss of both activation of *Il17a* transcription (Fig. 6F) and induction of Th17 differentiation (Fig. 6G). On the other hand, overexpression of c-Jun in the *Junb-*deficient CD4^+^ T cells partially restored IL-17A production, but less effective than that of JunB (Fig. 6E). Although c-Jun thus may have an ability to replace JunB in Th17 differentiation at least partially, JunB but not c-Jun plays the indispensable role, because c-Jun is expressed in Th17 cells to a much lesser extent than JunB (Fig. 5).

The *Fosl2*-encoded protein Fra2, another member of the AP-1 family, also has been reported to be important for Th17 cell specification^27^. Although JunB did not regulate expression of *Fosl2* during Th17 development (Supplementary Figure 9A), JunB was capable of forming a complex with Fra2 and activating *Il17a* transcription (Supplementary Figure 9B,C), suggesting that Fra2 may control Th17 differentiation by interacting with JunB.

### JunB is required for expression of *Rorc* and *Rora*

**Figure 7:**
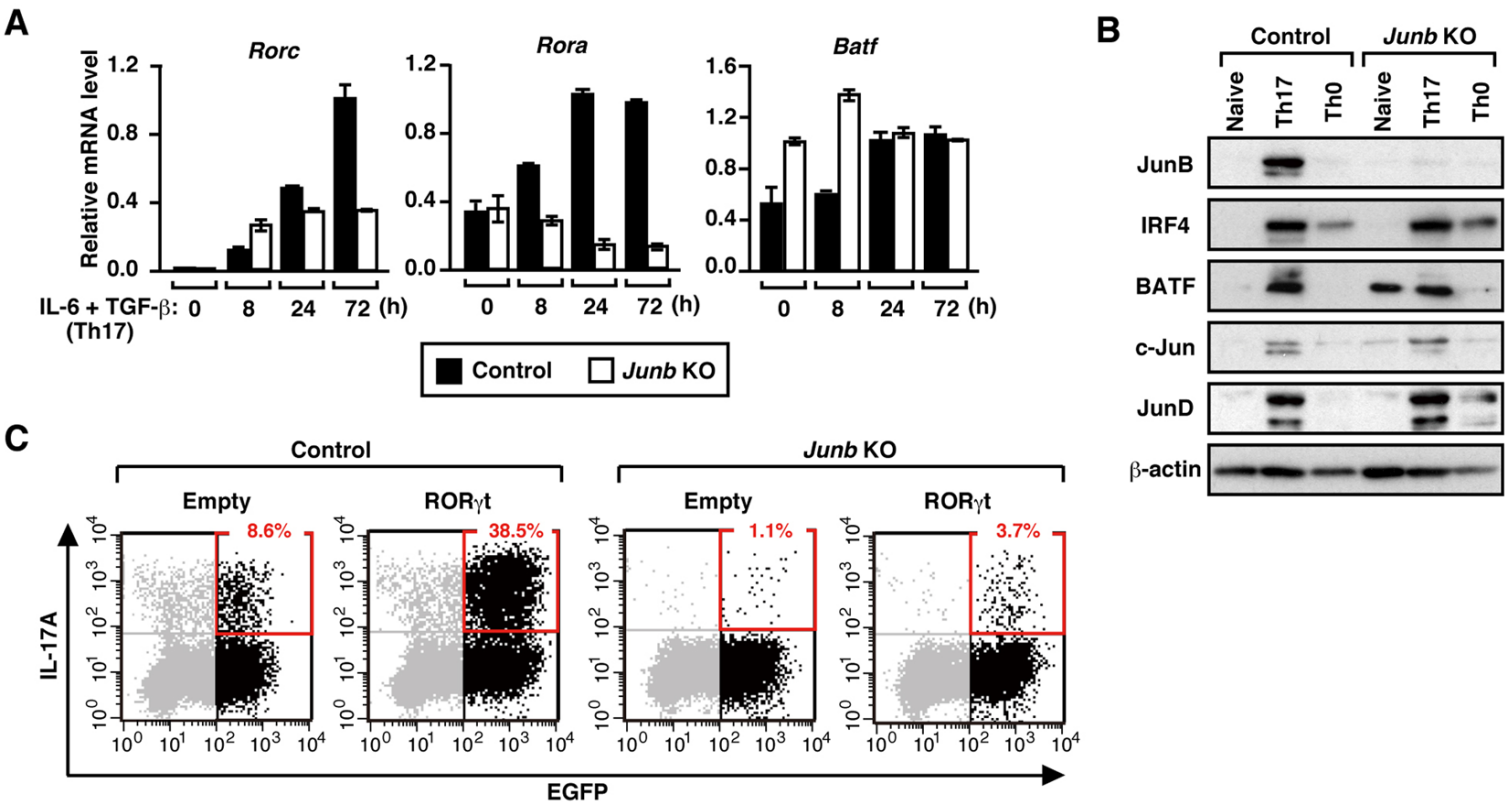
Expression of RORγt and RORα is impaired in *Junb*-deficient cells. (**A**) Real-time PCR analysis of *Rorc* and *Rora* expression in *Junb* ^f/+^ (control) and *Junb*-deficient (KO) CD4^+^ T cells cultured under Th17-polarizing conditions. Data are presented as mean ± SD. (**B**) Immunoblot analysis of production of IRF4, BATF, and Jun family proteins in CD4^+^ T cells cultured under the indicated conditions. (**C**) Bi-cistronic retroviral overexpression of RORγt together with EGFP and its effect on IL-17A production in CD4^+^ T cells cultured under Th17-polarizing conditions. IL-17A production was analyzed by flow cytometry; numbers indicate percent EGFP^+^ cells producing IL-17A.

The present findings indicate that JunB regulates Th17 development by forming a heterodimer with BATF. To further know the molecular mechanism whereby JunB functions, we tested its role in expression of the Th17 lineage-specifying factor RORγt (encoded by *Rorc*)^5,6^. It is known that *Rorc* expression under Th17-polarizing conditions is impaired in CD4^+^ T cells deficient in the Th17-polarizing transcription factors BATF^7^, IRF4^8^, and STAT3^9^, whereas the expression is not affected in CD4^+^ T cells lacking IκBζ (encoded by *Nfkbiz*), which also participates in Th17 development^13^. As shown in Fig. 7A, expression of *Rorc* mRNA was prevented in *Junb-*deficient CD4^+^ T cells at 24 and 72 h after stimulation. The RORγt-related protein RORα is also known to regulate development of Th17 cells, e.g., RORα and RORγt molecularly cross-compensate for Th17 differentiation^6^. Although expression of *Rora* was enhanced during Th17 differentiation in *Junb* ^f/+^ CD4^+^ T cells, the enhancement did not occur in *Junb*-deficient CD4^+^ T cells (Fig. 7A). The impaired expression of *Rorc* and *Rora* in *Junb*-deficient cells was confirmed by RNA-seq analysis (Fig. 2A). Furthermore, as shown in a chromatin immunoprecipitation analysis (Supplementary Figure 10), at around the transcription start site of *Rorc* and *Rora* as well as that of other Th17 signature genes, *Junb* deficiency resulted in deacetylation of histones H3 and H4, characteristic of transcriptionally inactive chromatin states. It has been reported that *Rora* expression is prevented also in *Batf*-deficient CD4^+^ T cells^7^ but not in *Nfkbiz*-deficient cells^13^. The reduced transcription of *Rorc* and *Rora* in *Junb-*deficient T cells does not seem to be due to perturbation in IRF4 and BATF, because the amounts of these transcription factors were not affected by *Junb* deficiency in Th17 cells (Fig. 7B). Of note, compensatory elevation in expression of c-Jun and JunD did not occur in the absence of *Junb* (Fig. 7B). Thus, JunB as well as BATF stimulates the expression of the ROR nuclear receptors RORγt and RORα, which likely contributes to differentiation of Th17 cells.

We next tested whether overexpression of RORγt is able to rescue impaired development of Th17 cells in *Junb*-deficient cells. Retroviral expression of RORγt restored IL-17A production impaired in *Junb-*deficient CD4^+^ T cells under Th17-polarizing conditions, but to a small extent (less than 10%) (Fig. 7C). Similarly, a loss of Th17 differentiation by ablation of *Batf* or *Irf4* is only slightly rescued by overexpression of RORγt^7,8^. The partial restoration indicates that RORγt expression is not sufficient for differentiation of Th17 cells, and thus raises a possibility that RORγt function is also supported by the Th17-polarizing factors JunB, BATF, and IRF4. It has recently been proposed that early binding of BATF and IRF4 to Th17-associated genes governs chromatin accessibility and subsequent recruitment of RORγt^27^, and thus it seems likely that the BATF-partner JunB also participates in initial chromatin accessibility of RORγt for Th17 cell specification.

RORγt-dependent expression of IL-17A is not only a feature of Th17 cells but also that of other RORγt^+^ cells including γδT cells^28^ and NKp46^−^CCR6^+^CD4^+^ group 3 innate lymphoid cells (ILC3)^29^. To know the role of JunB in IL-17A production by these cell types, we analyzed lymphocytes from short intestinal lamina propria of *Junb*-deficient and control mice. As expected, ablation of *Junb* led to a severe impairment in IL-17 production by Th17-containing populations such as CD4^+^TCRβ^+^ cells (Supplementary Figure 11A) and CD3ε^+^TCRγδ^−^ cells (Supplementary Figure 11B). By contrast, *Junb* depletion only modestly reduced IL-17A production by CD4^+^TCRβ^−^ cells, containing IL-17-producing ILC3 cells (Supplementary Figure 11A), and that by CD3ε^+^TCRγδ^+^ cells (γδT cells) (Supplementary Figure 11B). These findings indicate that JunB plays an indispensable role in IL-17A expression by Th17 cells, but not in that by ILC3 and γδT cells.

### JunB promotes Th17 differentiation by cooperating with BATF

Because JunB was capable of directly binding to not only BATF but also IκBζ (Supplementary Figure 1), it seemed possible that a functional link may exist between JunB and IκBζ. To test this possibility, we expressed IκBζ retrovirally in CD4^+^ T cells cultured under Th17-polarizing conditions. As previously reported^13^, overexpression of IκBζ or RORγt in wild-type CD4^+^ T cells increased a population of IL-17A-producing cells (Supplementary Figure 12A). Since the increase did not occur in *Junb*-deficient cells (Supplementary Figure 12B), IκBζ may possibly function in cooperation with JunB. However, expression of the Th17-specifying genes *Rorc* and *Rora* is dependent on JunB (Fig. 7A) but not on IκBζ, indicating that JunB does not cooperate with IκBζ. Furthermore, BATF effectively replaced IκBζ as a JunB-binding partner (Supplementary Figure 6C), and, unlike IκBζ, BATF regulates expression of both *Rorc* and *Rora* ^7^. These findings agree with the conclusion that JunB primarily functions by forming a productive dimer with BATF in Th17 development.

## Discussion

The present study provides genetic evidence that JunB is required for Th17 cell differentiation: *Junb*-deficient CD4^+^ T cells are defective in differentiating into Th17 cells, and *Junb*-deficient mice are refractory to induction of the Th17 cell-dependent autoimmunity EAE. The conclusion that JunB likely functions via forming an AP-1 complex with BATF, which is also indispensable for Th17 development, may be supported by recent observations in EMSAs using Th17 nuclear extracts: BATF can be complexed with JunB on AP-1-binding sites^7,24–26^. Although a part of such complexes also contains JunD^25^, JunD does not appear to make a major contribution to Th17 development. This is because JunD fails to activate Th17 signature genes in cooperation with BATF (Fig. 6). The role of c-Jun as a productive partner of BATF also seems to be minimal, since c-Jun is not abundantly expressed during differentiation into Th17 cells (Fig. 5) and compensatory elevation in the amount of c-Jun does not occur in the absence of *Junb* (Fig. 7).

The molecular mechanism whereby JunB controls Th17 differentiation likely involves JunB-dependent expression of the Th17 lineage-specifying factors RORγt and RORα; the expression is impaired by *Junb* ablation (Fig. 7). Consistent with this, the absence of the JunB-partner BATF also results in an impaired expression of *Rorc* and *Rora* ^7^. On the other hand, IκBζ, another JunB-binding protein, does not participate in expression of *Rorc* and *Rora* ^13^. The difference suggests that JunB does not function with IκBζ in Th17 differentiation, which is in agreement with the present observation that JunB interacts with BATF much more strongly with IκBζ (Supplementary Figure 6C). A BATF-containing AP-1 dimer has been shown to interact with IRF4 (or IRF8) on AP-1–IRF composite elements (AICEs), thereby activating a variety of genes that regulate development of immune cells such as Th2, Th17, B, and dendritic cells^15,24–27^. BATF in the AP-1 heterodimer and IRF4 are thus considered to cooperatively function in Th17 differentiation at least in part. Like JunB and BATF, IRF4 also regulates *Rorc* expression^8^. In addition to increased expression of *Rorc* and *Rora*, JunB appears to function also by repressing expression of Foxp3, a transcription factor that is known to inhibit Th17 cell differentiation by antagonizing RORγt function^14^. This is because Foxp3 expression is elevated in *Junb-*deficient cells under Th17-polarizing conditions (Fig. 3), which elevation is also observed in cells lacking the JunB-partner BATF^7^. Furthermore, since early binding of BATF to Th17-associated genes appears to govern chromatin accessibility and subsequent recruitment of RORγt^27^, it seems possible that the BATF-partner JunB also contributes to initial chromatin accessibility of RORγt for Th17 cell specification. This may explain at least partially the reason why forced expression of RORγt only partially rescues Th17 differentiation impaired in *Junb*-deficient cells (Fig. 7).

The present RNA-seq transcriptome analysis confirms the requirement of JunB for Th17 development: besides induction of Th17 signature genes, JunB turns on the Th17 gene expression program (Fig. 2). On the other hand, JunB deficiency only marginally affects gene expression in naive CD4^+^ T cells and Th0 cells (Fig. 2), which is consistent with the finding that CD44 and CD62L are normally expressed in *Junb*-deficient naive CD4^+^ T cells (Supplementary Figure 3E). The gene expression pattern of *Junb*-deficient CD4^+^ T cells under Th17-polarizing conditions falls into the same cluster as that of Th0 cells (Fig. 2C). Thus JunB is not involved in TCR-mediated conversion of naive helper T cells into Th0 cells.

The present study also demonstrates that *Junb-*deficient CD4^+^ T cells are able to differentiate into Th1, Th2, and Treg cells under the respective polarizing conditions (Fig. 3). Consistently, the JunB-binding protein BATF is not required for differentiation into these T cell subsets^7^. It has been reported that JunB protein level correlates with the extent of differentiation into Th2 cells^30–32^; accordingly, JunB is considered to facilitate Th2 cell development, although the direct effect of JunB has not been tested using *Junb*-knockout mice until the present study. Because the facilitation is mainly due to increased production of the autocrine factor IL-4 that is necessary for Th2 lineage commitment^30–32^, it seems likely that JunB is dispensable for Th2 differentiation in the presence of a high amount of IL-4, such as under the present Th2 polarizing conditions (Fig. 3). On the other hand, JunD has been reported to negatively regulate differentiation into Th2 cells^33^. Thus JunD appears to play a role distinct from that of JunB in Th2 differentiation as well as in Th17 differentiation.

As shown in the present study, JunB expression is elevated at the mRNA and protein levels during Th17 differentiation (Figs 1 and 5). It is known that the amount of JunB is regulated via various mechanisms. For example, JunB is stabilized at the protein level by CARMA1 (also known as CARD11), a scaffold protein exclusively expressed in lymphoid and myeloid cells^34^. Intriguingly, lack of CARMA1 selectively prevents Th17, but not Th1 or Th2 differentiation, and CARMA1-knockout mice are resistant to EAE^35^. The mechanism for CARMA1 in Th17 differentiation appears to be explained at least in part by the present conclusion that JunB plays a crucial role in Th17 differentiation. It has been also reported that serum glucocorticoid kinase 1 (SGK1) stabilizes JunB by preventing ubiquitination and degradation of this protein, which is mediated via the E3 ligase Nedd4-2 (also known as Itch) and its adaptor protein Ndfip^36^; and ablation of the SGK1 gene does not affect primary Th17 differentiation but attenuates IL-23R-mediated induction of pathogenic Th17 cells^37^. Thus, in addition to induction of Th17 differentiation, JunB may also contribute to the stabilization of the Th17 cell phenotype and pathogenicity acquisition. Control of JunB protein level at various steps would provide new therapeutic opportunities for human inflammatory autoimmune diseases in which Th17 cells have been implicated, such as psoriasis and multiple sclerosis^38,39^.

## Materials and Methods

### Mice

*Junb* ^f/f^ mice were generated according to the standard technique^40,41^. The BAC (bacterial artificial chromosome) clones containing mouse *Junb* gene (C57BL/6J) were obtained from BACPAC resource (Children’s Hospital Oakland Research Institute). The targeting vector was constructed in pBluescript (Stratagene) to replace the exon of the *Junb* gene with the floxed *Junb* gene containing PGK-*neo* ^r^ (neomycin phosphotransferase gene driven by phosphorglycerate kinase promoter) cassette for positive selection and diphtheria toxin cassette for negative selection. A linearized targeting vector was electroporated into MS12 ES cells derived from blastocysts of C57BL/6J mice^42^. Positive clones were evaluated by Southern blot analysis with probes specific for 5′- and 3′-ends of the recombination site (5′-probe (600 nt) and 3′-probe (493 nt)) and also with an internal probe (475 nt). The confirmed clones were injected into blastocysts. The blastocysts were implanted into the uterus of a pseudo-pregnant mouse to yield chimeric mice. By crossing the chimera mice to C57BL/6 mice, the mice harboring the floxed *Junb* and PGK-*neo* ^r^ allele were generated. The correct recombination in F2 mice was confirmed by Southern blot analysis using the probes mentioned above and also by DNA sequencing around the recombination site. To remove PGK-*neo* ^r^ cassette, the flippase expression vector (pCAGGD-FLPe, Gene Bridges) was injected into the fertilized eggs obtained from mating wild-type C57BL/6 mice with the mice harboring the floxed *Junb* and PGK-*neo* ^r^ allele^43^. The removal of PGK-*neo* ^r^ cassette by flippase was confirmed by Southern blot analysis^42,44,45^ using the internal probe (475 nt) and also by PCR with primers (mJb-F30, 5′-ATGACCCATGTCAGCAACGG-3′; mJb-R22, AAGTGCGTGTTTCTTCTCCACAG). *Junb* ^f/+^ mice were backcrossed onto wild-type C57BL/6 mice more than 15 times to eliminate the potential mutations caused during the gene manipulation. *Junb* ^f/f^ mice were obtained by mating *Junb* ^f/+^ mice; the obtained pups had normal Menderian distribution. *Junb* ^f/f^ mice also appeared to develop normally. *Junb* ^f/f^ mice were crossed to *Meox2* ^+/Cre^ mice^46^ (The Jackson laboratory) to obtain *Junb* ^f/+^; *Meox2* ^+/Cre^ mice. These mice were then crossed with *Junb* ^f/+^ or *Junb* ^f/f^ mice for generation of *Junb*-deficient (*Junb* ^f/f^; *Meox2* ^+/Cre^) mice. The loss of the *Junb* gene by Cre-mediated recombination was verified by PCR using the following primers: 5′-CTGACAATTCCAGTTCTTTAAGC-3′, 5′-ATGACCCATGTCAGCAACGG-3′, and 5′-AAGTGCGTGTTTCTTCTCCACAG-3′. All animals were housed and maintained in a specific pathogen-free animal facility at Kyushu University. All experiments were performed in accordance with the guidelines for Proper Conduct of Animal Experiments (Science Council of Japan). The experimental protocol was approved by the Animal Care and Use Committee of Tokyo Metropolitan Institute of Gerontology, Kyushu University (Permit Numbers: A22-005, A24-042, and A26-102), and Toho University School of Medicine (17-43-288). All efforts were made to minimize the number of animals and their suffering.

### ***In vitro*** differentiation of CD4^+^ T cells

Naive CD4^+^ T cells were prepared from the spleen and lymph nodes of 6–9 week old mice by magnetic sorting using Dynabeads^®^ Mouse CD4 (L3T4, Invitrogen) and DETACHaBEAD^®^ Mouse CD4 (Invitrogen), as described previously^47^. The CD4^+^ T cells were activated for 3 days via TCR with plate-bound 5 μg/ml of an anti-CD3ε antibody (145-2C11, BioLegend) and soluble 2.5 μg/ml of an anti-CD28 antibody (35.71, BioLegend) under the following differentiation conditions: 10 μg/ml of an anti-IFN-γ antibody (XMG 1.2, BioLegend) and 10 μg/ml of an anti-IL-4 antibody (11B11, BioLegend) for Th0; 10 μg/ml of the anti-IFN-γ antibody, 10 μg/ml of the anti-IL-4 antibody, 40 ng/ml of mouse IL-6 (Peprotech), and 4 ng/ml of human TGF-β1 (R&D) for Th17; 10 μg/ml of the anti-IL-4 antibody and 40 ng/ml of mouse IL-12 p70 (BioLegend) for Th1; 10 μg/ml of the anti-IFN-γ antibody and 40 ng/ml of mouse IL-4 (Peprotech) for Th2; 10 μg/ml of the anti-IFN-γ antibody, 10 μg/ml of the anti-IL-4 antibody, 4 ng/ml of human TGF-β1 for Treg.

### Cell surface staining and intracellular cytokine staining

For cell surface staining, naive CD4^+^ T cells were stained with fluorescence-labeled antibodies: fluorescein isothiocyanate (FITC)-labeled anti-mouse CD8a (53–6.7, BioLegend), phycoerythrin (PE)-labeled anti-CD4 (RM4-5, BioLegend), allophycocyanin (APC)-labeled anti-CD4 (RM4-5, TONBO), APC-labeled anti-CD3ε (145-2C11, TONBO), APC/Cy7-labeled anti-CD45.2 (clone104, BioLegend), PE/Cy7-labeled anti-TCRβ (H57-597, TONBO), PE/Cy7-labeled anti-TCRγ/δ (GL3, BioLegend), FITC-labeled anti-mouse CD62L (MEL-14, BioLegend), PE/Cy5-labeled anti-mouse/human CD44 (1M7, BioLegend), FITC-labeled anti-mouse Ly6G (1A8, TONBO), and PE-labeled anti-mouse CD11b (M1/70 TONBO) antibodies were used. For intracellular cytokine staining, cells were stimulated for 4 h with phorbol 12-myristate 13-acetate (PMA) (50 ng/ml) and ionomycin (500 ng/ml) in the presence of GolgiPlug (BD Bioscience), and then fixed in a buffer containing 4% paraformaldehyde (BioLegend). The fixed cells were permeabilized in a permeabilization buffer (BioLegend) and stained with PE-labeled anti-mouse IL-17A (TC11-18H10, BD Pharmingen) and FITC-labeled anti-IFN-γ (XMG1.2, BioLegend) antibodies. Flow cytometry was carried out using FACS Calibur (BD Bioscience) at the Research Support Center, Research Center for Human Disease modeling, Graduate School of Medical Sciences, Kyushu University or LSR Fortessa X-20 cell analyzer (BD Bioscience).

### Induction of EAE and histological analysis

The synthetic peptide MOG_35–55_ (MEVGWYRSPFSRVVHLYRNGK) was purchased from Scrum Inc. EAE was induced by subcutaneous immunization with MOG_35–55_ in complete Freund’s adjuvant and intraperitoneal injection of pertussis toxin, according to the method of Langrish *et al*.^48^. On day 0, mice were subcutaneously immunized with MOG_35–55_ (200 μg per mouse) emulsified in complete Freund’s adjuvant supplemented with 500 μg per mouse of *Mycobacterium tuberculosis* H37RA (Difco). Pertussis toxin (500 ng per mouse, Calbiochem) was intraperitoneally injected on day 0 and day 2. Disease severity was scored based on the EAE clinical signs as follows: 0, no clinical sign; 1, tail limpness; 2, hind limb weakness; 3, hind limb paralysis; 4, fore limb weakness; 5, quadriplegia; 6, death. For myelin staining, freshly prepared spinal cords were fixed in 4% paraformaldehyde overnight, and embedded in paraffin. Tissue sections (5 μm) were rehydrated and stained with Luxol fast blue. For immunohistochemical analysis, spinal cords were fixed in 4% paraformaldehyde on ice for 1 h, embedded in Tissue-Tek OCT compound (Sakura Fineteck), and frozen at −80 °C. Cryosections (10 μm) were blocked for 1 h with PBS containing 1% BSA, and stained with FITC-conjugated anti-mouse CD3ε (145-2C11, BD Biosciences) and PE-conjugated anti-mouse CD11b (M1/70, BD Biosciences) antibodies, and 4′,6-diamidino-2-phenylindole (DAPI) (DOJINDO).

### RNA-seq and bioinformatic analysis

Two and a half micrograms of total RNA was subjected to ribosomal RNA depletion using Ribo-Zero Magnetic Gold Kit (Human/Mouse/Rat) (Epicentre) followed by RNA-seq library preparation using NEBNext Ultra Directional RNA Library Prep Kit for Illumina (NEB) according to the manufacturer’s instructions. Each library was quantified using Kapa Library Quantitation Kit (Kappa) and sequenced on Illumina HiSeq. 2500 to generate 101-bp paired-end reads. Raw FASTQ reads were trimmed for adapter sequences and low-quality reads using Trimmomatic version 0.32^49^. Trimmed reads were then aligned to the mouse reference genome mm9 and the transcriptome defined by the mm9 genes.gtf table obtained from the UCSC Genome Browser using TopHat version 2.0.13^50^. Mapped reads were assigned to all exons using featureCounts^51^. The differential analysis was conducted using the Bioconductor package edgeR^52^, applying trimmed Mean of M-values library normalization. A gene was defined as differentially expressed, if the false discovery rate (FDR) corrected with Benjamini-Hochberg method was less than 0.001 and if the log2 fold change (log2FC) was more than 1 (up-regulated) or less than −1 (down-regulated). Differentially expressed genes were subjected to pathway enrichment analysis using Ingenuity Pathway Analysis (Qiagen). Over-represented pathways were defined as those with Benjamini-Hochberg–corrected P values of Fisher’s exact test less than 0.01. The same gene set was also analyzed using ConsensusPathDB^53^, which enables an integrative usage of widely used pathway databases, namely KEGG, Pathway Interaction Database, Reactome, and Wikipathways. To exploit the latest human pathway datasets (Release 32, 2017) of ConsensusPathDB, human orthologues of the differentially expressed genes were identified with HomoloGene (https://www.ncbi.nlm.nih.gov/homologene) and Mouse Genome Informatics (www.informatics.jax.org) and used as a query to find enriched pathways. FPKM values were used for unsupervised hierarchical clustering based on the Spearman correlation distance and the Ward’s linkage clustering algorithm with the hclust function implemented in R software.

### Cell culture

The RAW264.7 mouse macrophage-like cells, the HEK293T human embryonic kidney cells, and the NIH3T3 mouse fibroblasts were cultured in Dulbecco’s modified Eagle’s medium supplemented with 10% fetal bovine serum, penicillin (100 units/ml), and streptomycin (100 µg/ml). The BW5147α^−^β^−^ mouse thymoma cells were cultured in RPMI medium supplemented with 10% fetal bovine serum, penicillin (100 units/ml), and streptomycin (100 µg/ml).

### Mass spectrometric analysis of IκBζ-binding proteins

Modified RAW264.7 cells expressing FLAG–IκBζ under the control of the zinc-inducible sheep metallothionein Ia promoter^11^ were stimulated with 50 nM ZnSO_4_ for 3 h and subsequently with LPS (100 ng/ml) for 2 h. Nuclei prepared from the stimulated cells were sonicated in a buffer containing 140 mM KCl, 0.5% NP-40, 5% glycerol, and 20 mM HEPES, pH 8.0; the sonicates were subjected to immunoprecipitation using anti-FLAG (M2) antibody-conjugated agarose (Sigma). After washing with PBS (137 mM NaCl, 2.68 mM KCl, 8.1 mM Na_2_HPO_4_, and 1.47 mM KH_2_PO_4_, pH 7.4) containing 0.1% Triton X-100, the precipitated proteins were separated by SDS–PAGE (10%), followed by staining with *Coomassie Brilliant Blue* (CBB). Protein identification using liquid chromatography and tandem mass spectrometry (LC-MS/MS) was performed at the Laboratory for Technical Support, Medical Institute of Bioregulation, Kyushu University. Bands separated by SDS–PAGE were excised from the gel, and subjected to reduction with DTT, *S*-carbamidomethylation with iodoacetamide, and in-gel digestion with trypsin. Fragmented peptides were analyzed by LC-MS/MS as previously described^54^. The analysis reproducibly identified JunB in addition to the NF-κB p50 subunit, a well-known partner of IκBζ^10–12^.

### An ***in vitro*** pull-down binding assay

Maltose-binding protein (MBP)-fusion proteins were expressed in *E. coli* BL21 strain and purified using Amylose Resin (New England Biolabs). FLAG-tagged proteins were synthesized *in vitro* using TNT^®^ T7 Quick for PCR (Promega). The MBP-fusion protein bound to the resin was mixed with the FLAG-tagged protein in a binding buffer (150 mM NaCl, 5% glycerol, 1 mM DTT, 1 mM EDTA and 25 mM Tris-Cl, pH 8.0) and incubated for 1 h at 4 °C. After washing with the binding buffer containing 0.05% Triton X-100, proteins were eluted with 20 mM maltose. The eluate was subjected to SDS–PAGE, followed by staining with CBB or by immunoblot analysis with the anti-FLAG antibody (M2, Sigma).

### Activation of CD4^+^ T cells prepared from OT-II TCR transgenic mice

CD4^+^ T cells prepared from OT-II TCR transgenic mice, which express TCR specific for chicken ovalbumin (OVA)^22,55^ were co-cultured for 3 days with the OVA_323–339_ peptide (25 μg/ml) in the presence of APCs; APCs were prepared from splenocytes by depletion of T cells using CD90.2 MicroBeads (Miltenyi Biotec), followed by γ-irradiation (30 Gy).

### Retroviral transduction

cDNAs for RORγt, IκBζ, JunB, c-Jun, and JunD were cloned into the bicistronic retroviral vector pMXs-IG^56^, which expresses EGFP under the control of an internal ribosomal entry site. The Plat-E packaging cells^57^ were transfected with the retroviral vector and cultured for 2 days. The culture supernatant was filtrated using a surfactant-free cellulose acetate filter (0.45-μm, Sartorius). Activated CD4^+^ T cells were infected with the retroviral supernatant on days 1, 2 and 3 by centrifugation at 650 × *g* for 2 h at 32 °C in the presence of polybrene (10 μg/ml). On day 4, IL-17 production was measured by intracellular cytokine staining.

### Co-immunoprecipitation and immunoblot analysis

HEK293T cells were transfected with pcDNA3 encoding FLAG-tagged Jun family proteins and haemagglutinin (HA)-tagged BATF. After 24 h, the FLAG-tagged protein was immunoprecipitated using anti-FLAG (M2)-conjugated agarose (Sigma) and analyzed by immunoblot with the anti-FLAG (M2, Sigma) and anti-HA (16B12, Covance) antibodies. Endogenous proteins in T cell lysates were analyzed by immunoblot with anti-IRF4 (sc-6059, Santa Cruz), anti-BATF (sc-100974, Santa Cruz), anti-JunB (sc-46, Santa Cruz), anti-c-Jun (sc-1694 and sc-74543, Santa Cruz), anti-JunD (sc-74, Santa Cruz), and anti-β-actin (sc-47778, Santa Cruz) antibodies.

### Estimation of relative amounts for endogenous Jun family proteins in Th17 cells

HEK293T cells were transfected with pcDNA3 encoding FLAG-tagged JunB, c-Jun, or JunD to express substantial amounts of these proteins. FLAG–JunB, FLAG–c-Jun or FLAG–JunD in the cell lysates were used as standard proteins in immunoblot analysis for estimation of relative amounts of endogenous JunB, c-Jun, and JunD in Th17 cells. The same amounts of FLAG–JunB, FLAG–c-Jun or FLAG–JunD, estimated by immunoblot using the anti-FLAG antibody (M2, Sigma), were serially diluted and subjected to immunoblot analysis; in the analysis, antibodies specific for JunB (sc-46, Santa Cruz), c-Jun (sc-74543, Santa Cruz), or JunD (sc-74, Santa Cruz) were used for estimation of the corresponding endogenous Jun protein in the Th17 cell lysate (see Fig. 4a). These primary antibodies were probed with the fluorescently labeled secondary antibody, anti-mouse IRDye800CW-conjugated (LI-COR Biosciences). Detection and quantification were performed using the Odyssey Infrared Imaging System (LI-COR Biosciences)^58,59^. Standard curves were generated by plotting the band intensities against the amounts of FLAG-tagged Jun-family proteins, and used for calculation of the relative expression level of endogenous JunB, c-Jun, and JunD in Th17 cells (see supplemental Fig. 5).

### Quantitative reverse transcription (RT)–PCR and ELISA

Extraction of total RNA, reverse transcription and real-time quantitative PCR were performed as described previously^11^. The sequences of the primers used for quantitative PCR are as follows: *Hprt*, 5′-GCAGTACAGCCCCAAAATGG-3′ and 5′-AACAAAGTCTGGCCTGTATCCAA-3′; *Il17a*, 5′-CTGGAGGATAACACTGTGAGAG-3′ and 5′-TGCTGAATGGCGACGGAGTTC-3′; *Il17f*, 5′-CAAAACCAGGGCATTTCTGT-3′ and 5′-ATGGTGCTGTCTTCCTGACC-3′; *Il21*, 5′-AGCCCCCAAGGGCCAGATCGC-3′ and 5′-AGCTGCATGCTCACAGTGCCCCTTT-3′; *Il23r*, 5′-CCCAGACAGTTTCCCAGGTTACAGC-3′ and 5′-TGGCCAAGAAGACCATTCCCGACA-3′; *Rorc*, 5′-CCGCTGAGAGGGCTTCAC-3′ and 5′-TCCCCACAGATCTTGCAAGG-3′; *Rora*, 5′-GAACACCTTGCCCAGAACAT-3′ and 5′-AGCTGCCACATCACCTCTCT-3′; *Foxp3*, 5′-GGCGAAAGTGGCAGAGAGG-3′ and 5′-AAGGCAGAGTCAGGAGAAGTTG-3′; *Batf*, 5′-CCAGAAGAGCCGACAGAGAC-3′ and 5′-GAGCTGCGTTCTGTTTCTCC-3′; *Jun*, 5′-GCCTACGGCTACAGTAACCC-3′ and 5′-GAGAAGGTCCGAGTTCTTGG-3′; *Junb*, 5′-CTGAAACCCACCTTGGCGC-3′ and 5′-CCGAAAAGTAGCTGCCTGCC-3′; *Fosl2*, 5′-GACTCCCAGGTGGGAGTGC-3′ and 5′-CAGATAGGAGGAGTCTGAGG-3′; *Acp5*, 5′-CTGCTTGTCCGCTAACGGG-3′ and 5′-TCTGCCAGAGTACCAGGGC-3′; *Mmp9*, 5′-CTCTCTACTGGGCGTTAGGG-3′ and 5′-AGAGGAGTCTGGGGTCTGG-3′; *Defb4*, 5′-AACCAGCAAGATGAATAAATTGC-3′ and 5′-TGTGCATCCCCTAGAACTGG-3′; *Il23a*, 5′-GGGGAACATTATACTTTCCTGG-3′ and 5′-CTAGATTCTGTTAGAACTGAGG-3′; *S100a9*, 5′-ATCTTTGCCTGTCATGAGAAGC-3′ and 5′-GTTGGGCAGCTGTCACATGG-3′; I*l19*, 5′-GCCAACTCTTTCCTCTGCGT-3′ and 5′-GGTGGCTTCCTGACTGCAGT-3′; and *Il24*, 5′-TTGGATGCTCCGACTGACC-3′ and 5′-TACGGTTTCCAGGGAAGGTG-3′. Expression level of mRNA was normalized to that of *Hrpt*. For ELISA, CD4^+^ T cells cultured under Th0-, Th1-, Th2-, and Th17-polarizing conditions were stimulated for 24 h with PMA (50 ng/ml) and ionomycin (500 ng/ml). Protein concentrations for IL-17A, IFN-γ and IL-4 in the culture supernatant were measured using ELISA MAXTM Deluxe Set Mouse IL-17A, IFN-γ, and IL-4 (BioLegend), respectively.

### Imiquimod-induced model of psoriasis-like skin inflammation

Psoriasis-like dermatitis was induced by treatment of mice with imiquimod, according to the method by Yoshiki *et al*.^60^. Mice were topically treated with a daily dose of 50 mg of imiquimod cream (5%) (Mochida Pharmaceutical) on one ear for 5 consecutive days. Severity of the dermatitis was quantified by the extent of ear swelling and by the induction level of psoriasis-associated genes in samples obtained by ear biopsy. Ear thickness was measured daily with a Mitutoyo digmatic micrometer (Mitsutoyo). For estimation of psoriasis-associated gene expression, total RNA was extracted with TRIsure (BIOLINE) using Micro Smash MS-100 (TOMY), followed by RT-qPCR analysis. Expression level of mRNA was normalized to that of *Hrpt*.

### Luciferase reporter Assay

A genomic DNA fragment corresponding to the 5′-upstream region of murine *Il17a* (−6,971/+56), *Il17f* (−2,107/+71), or *Il23r* (−1,540/+75) was amplified by PCR and inserted into pGL3-basic vector (Promega). HEK293T, NIH3T3, or BW5147α^−^β^−^ cells were transfected using X-tremeGENE HP DNA transfection Reagent (Roche) with pcDNA3 encoding a FLAG-tagged Jun family proteins (c-Jun, JunB, or JunD), pcDNA3-FLAG–BATF, the reporter plasmid containing the promoter of *Il17a*, *Il17f*, or, *Il23r*, and the internal control plasmid pRL-TK (Promega). After cultured for 18 h (NIH3T3 and BW5147α^−^β^−^ cells) or 24 h (HEK293T cells), luciferase activities were measured using the Dual-Luciferase Reporter Assay System (Promega).

### Chromatin immunoprecipitation (ChIP)

ChIP assays were performed as previously described^11^. Briefly, formaldehyde-fixed chromatin was subjected to sonication to obtain DNA fragments ranging in size from 100 to 400 bp. Chromatin fragments were immunoprecipitated using anti-acetylated histone H3 antibody (Millipore, #06-599), or anti-acetylated histone H4 antisera (Millipore, #06-866), or rabbit control Immunoglobulin (DAKO). After reversal of formaldehyde crosslinks, precipitated DNA was analyzed by quantitative real-time PCR using primers as follows: *Il17a* promoter, 5′-CACCTCACACGAGGCACAAG-3′ and 5′-ATGTTTGCGCGTCCTGATC-3′; *Il17f* promoter, 5′-GGCTGCTTCTTCCCTCAGG-3′ and 5′-TAAAACTGACAGGTACTACTGC-3′; *Il21* promoter, 5′-CTGCAATGGGAGGGCTTGG-3′ and 5′-CTTCAACCTGACTGTGCACAG-3′; *Il23r* promoter, 5′-CAAGAGTCCTTAAAACCCACC-3′ and 5′-CATGGGAAGTGGCATTATTAGG-3′; *Rorc* (RORγt) promoter, 5′-CAGAAACACTGGGGGAGAGC-3′ and 5′-ACACAGCTGGCAGTGGAGG-3′; *Rora* (RORα isoform 4) promoter, 5′-GCAAGGCAGAGAGCTTCCG-3′ and 5′- CACCAAAGTCCCTCGCCAC-3′; *Il5* 3′-end, 5′-ATGAGAGGATGAATGAATGAATG-3′ and 5′-AGCTCTTCATCCTTGTACAGC-3′; *Actb* promoter, 5′-CTGTGGCGTCCTATAAAACCC-3′ and 5′-CGAAGGAGCTGCAAAGAAGC-3′.

### Osteoclast differentiation

Differentiation of osteoclasts from bone marrow cells was performed as reported by Matsubara *et al*.^61^. Bone marrow cells were cultured in the presence of M-CSF (10 ng/ml) and sRANKL (50 ng/ml) for 5 days, and analyzed by a tartrate-resistant acid phosphatase (TRAP) assay using Acid Phosphatase, Leukocyte (TRAP) kit (Sigma) or by qPCR for quantifying expression of the osteoclast marker genes *Acp5* and *Mmp9* ^20^.

### Preparation of lymphocytes from short intestinal lamina propria

Lymphocytes were prepared from short intestinal lamina propria as described by Satoh-Takayama *et al*.^62^ with minor modifications. Briefly, after removal of epithelia with 1.0 mM EDTA, fragments of the small intestine were digested with collagenase (1 mg/ml, Wako). The tissues suspended in a 40% Percoll solution (GE Healthcare) were underlaid with an 80% Percoll solution. Following centrifugation for 20 min at 880 × *g*, cells at the 40%–80% interface were harvested.

### Knockdown using short interfering RNA (siRNA)

Knowkdown using siRNA in CD4^+^ T cells were performed as described by Ciofani *et al*.^27^. CD4^+^ T cells from wild-type mice were cultured for 16 h under Th0 conditions, and 300 pmol of control or c-Jun siRNA (Invitrogen) was introduced using the Mouse T cell Nucleofector Kit (Lonza) according the manufacture’s instruction. After recovery by incubation in fully supplemented culture media (Lonza), the cells were cultured for 3 days under Th17 conditions. Sequences for c-Jun siRNAs are as follows: #1, 5′-UACUGUAGCCGUAGGCACCGCUCUC-3′; #2, 5′-AUGACUUUCUGCUUAAGCUGUGCCA-3′; #3, 5′-AAACGUUUGCAACUGCUGCGUUAGC-3′.

### Statistical analysis

Statistics are specified in the figure legends. For EAE experiments, the number of animals used is described as the *n* value in the figure legend. Statistical significance between two means was assessed with an unpaired, two-sided Student’s *t* test.

## Electronic supplementary material

Supplementary information
