# Supplementary material for: The AP-1 transcription factor JunB is required for Th17 cell differentiation
Source: Sci Rep. 2017 Dec 12;7:17402. doi: 10.1038/s41598-017-17597-3 (PMC5727176; doi:10.1038/s41598-017-17597-3)
Supplement: Supplementary file 1 — Supplementary information [file 41598_2017_17597_MOESM1_ESM.pdf]

Supplementary Information

The AP-1 transcription factor JunB is required for Th17 cell differentiation

Soh Yamazaki, Yoshihiko Tanaka, Hiromitsu Araki, Akira Kohda, Fumiyuki Sanematsu, Tomoko Arasaki, Xuefeng Duan, Fumihito Miura, Takaharu Katagiri, Ryodai Shindo, Hiroyasu Nakano, Takashi Ito, Yoshinori Fukui, Shogo Endo, and Hideki Sumimoto

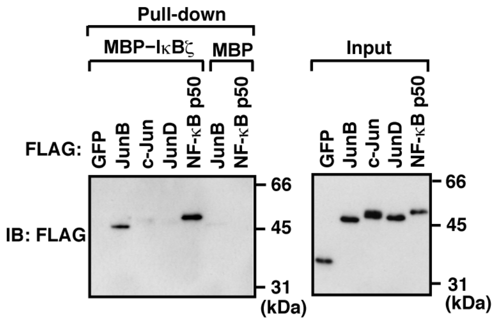

Supplementary Figure 1. Interaction of JunB and IκBζ. Bacterially expressed MBP–IκBζ and *in vitro* translated FLAG–JunB were mixed and pulled down with Amylose Resin, followed by immunoblot (IB) analysis with anti-FLAG antibody.

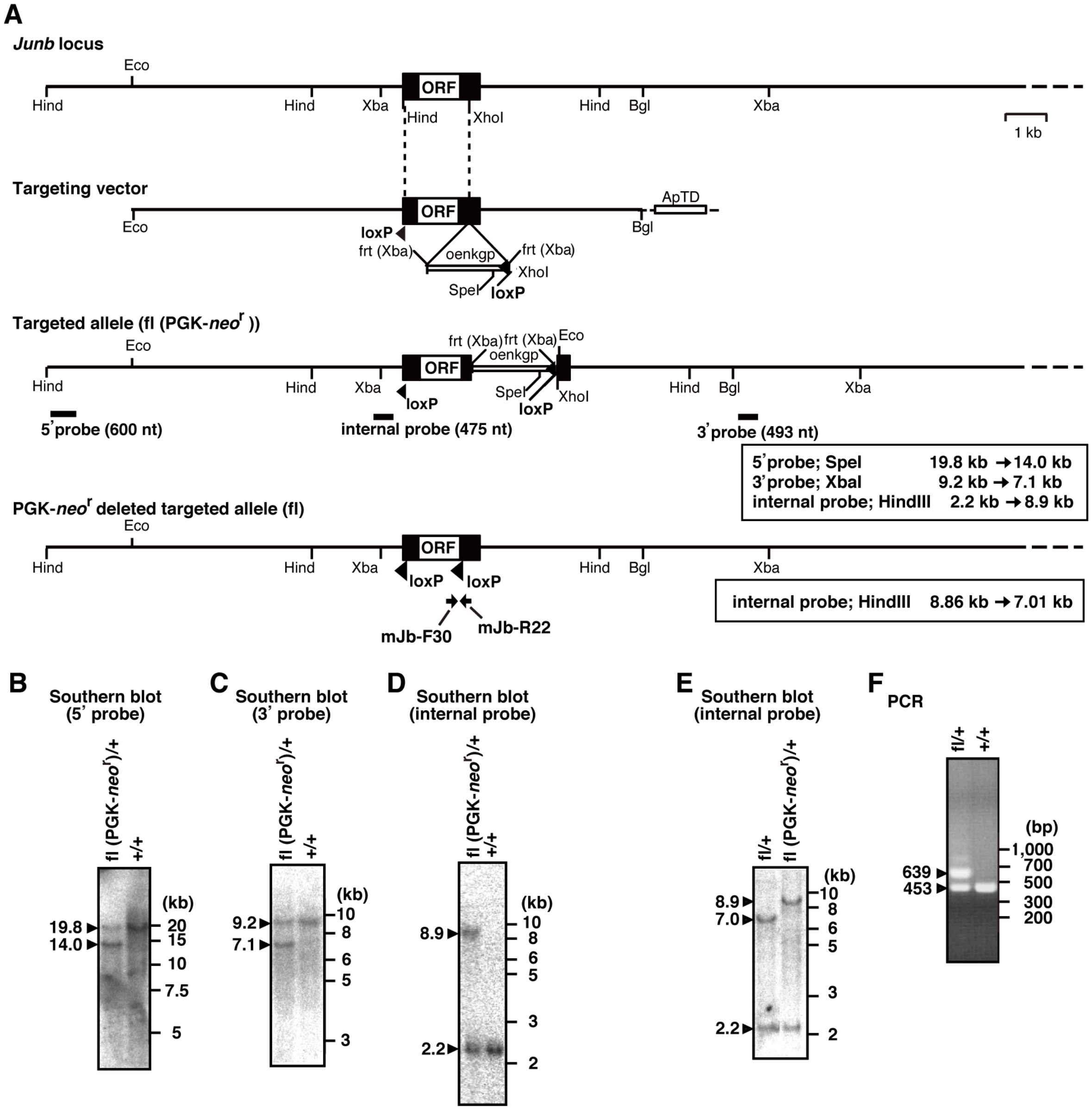

Supplementary Figure 2. Generation of *Junb*$^{f/f}$ mice by homologous recombination and characterization of naive *Junb*-deficient CD4^{+} T cells. (A) Gene structure of the mouse endogenous *Junb* locus, the targeting vector used, and the targeted mutant alleles are shown. *Junb* of a single exon was replaced with the targeting vector containing the *Junb* exon, PGK-*neo*^{r} cassette and loxP for the conditional deletion by homologous recombination in ES cells derived from C57BL/6 mice. To remove the PGK-*neo*^{r} cassette, the flippase expression vector was injected into the fertilized eggs obtained from mating wild-type mice with the mice harboring the

floxed *Junb* and PGK- *neo*^{r} allele. The yielded floxed *Junb* locus is shown in the bottom. PGK-*neo*^{r}, the neomycin phosphotransferase gene driven by phosphoglycerine kinase promoter; DTpA, the diphtheria toxin cDNA with poly(A)$^{+}$ sequence; frt, the sequence recognized by Flp DNA recombinase; loxP, the sequence recognized by Cre DNA recombinase; ORF, open reading frame for *Junb*. Restriction enzymes: Hind, *Hind*III; Eco, *Eco*RI; Xba, *Xba*I; XhoI, *Xho*I; Bgl, *Bgl*II. (B, C and D) Confirmation of homologous recombination in the *Junb* locus by Southern blot analysis. Restriction digested genome DNA from the mice was subjected to Southern blot hybridization analysis. The band shifts attributable to the homologous recombination were confirmed by probing the blot with the ^{32}P-labeled 5' -, 3'-, or internal probe. The expected band shifts were boxed in (A). (E) The removal of the PGK-*neo*^{r} cassette was confirmed by Southern blot using the internal probe. The expected band shift is indicated in bottom box in (A). (F) Confirmation of the removal of PGK-*neo*^{r} cassette by PCR analysis using the primers mJb-F30 and mJb-R22.

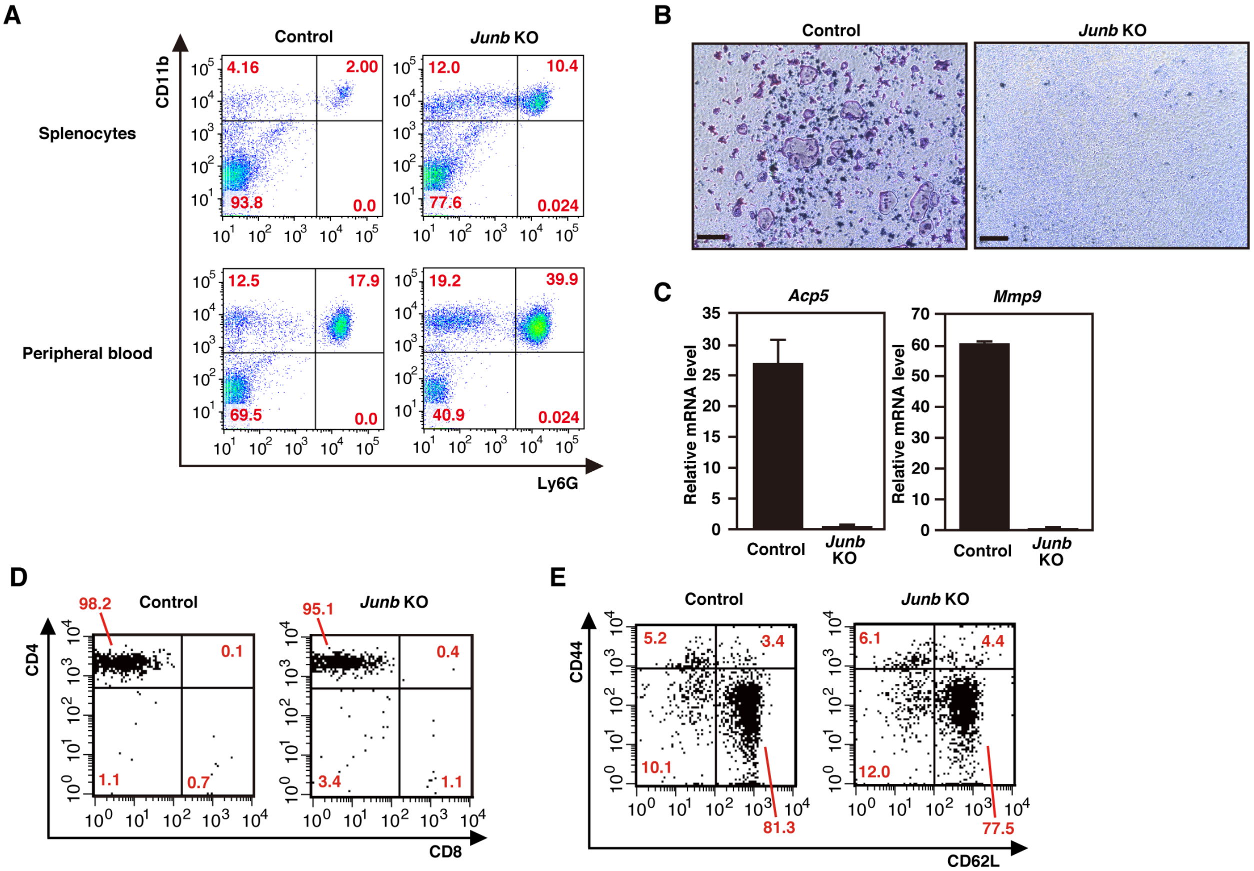

Supplementary Figure 3. Normal development of naive CD4$^+$ T cells in *Junb*-deficient mice. (A) Splenocytes and peripheral blood cells were prepared from *Junb*$^{f/+}$ (control) and *Junb*-deficient (KO) mice, and cell surface expression of Ly6G and CD11b was analyzed by flow cytometry. (B, C) Bone marrow cells from control and *Junb*-deficient mice were cultured in the presence of M-CSF and RANKL for differentiation of osteoclasts. The differentiation was analyzed by TRAP assay (B) or qPCR for quantifying expression of marker genes (C). Scale bars in (B), 400 μm. (D and E) Naive CD4$^+$ T cells were prepared from the spleen and lymph nodes of control and *Junb*-deficient mice, and cell surface expression of CD4 and CD8 (C) or that of CD62L and CD44 (D) was analyzed by flow cytometry.

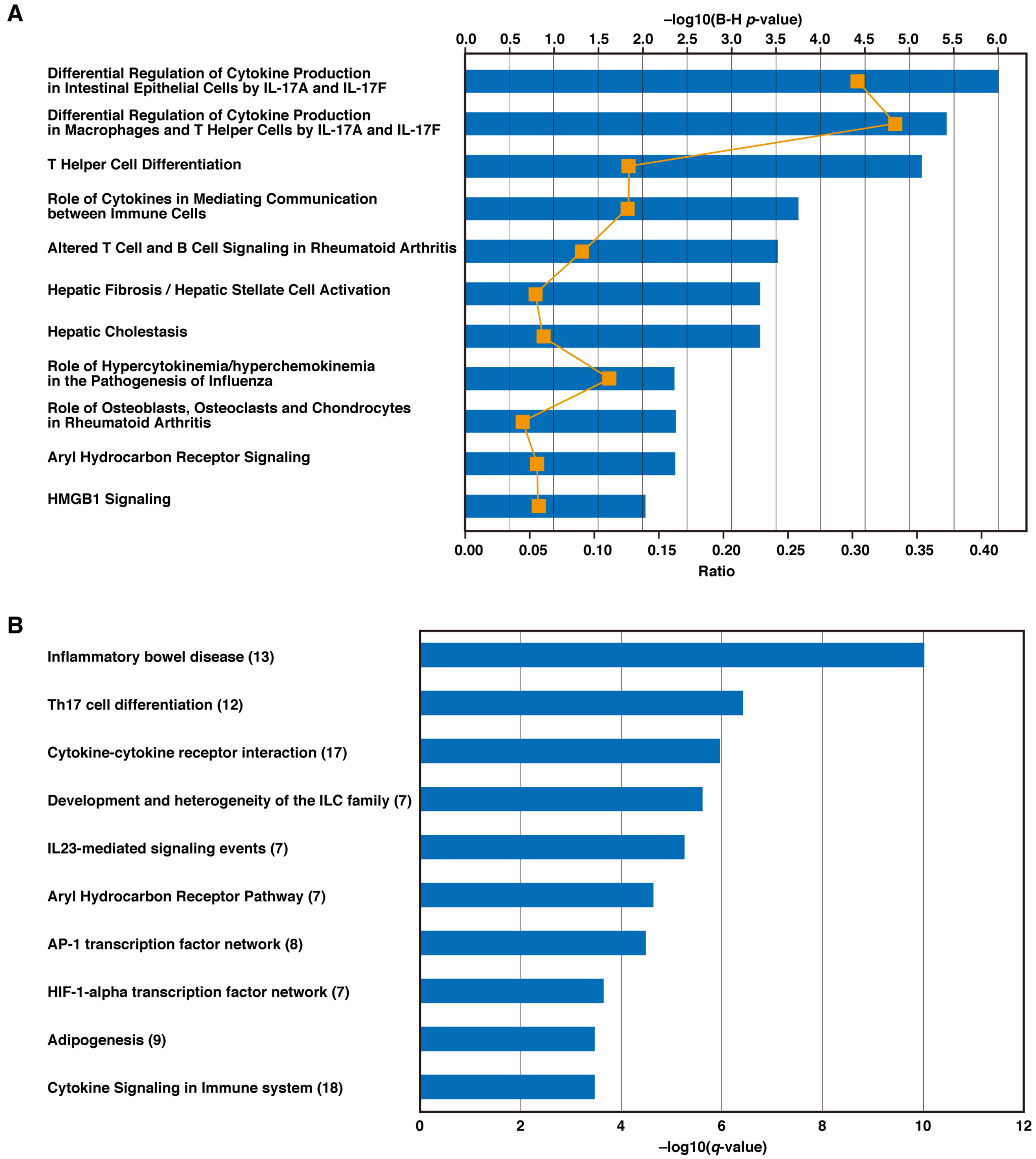

Supplementary Figure 4. Pathway enrichment analysis. (A) Ingenuity Pathway Analysis of 234 genes down-regulated in *Junb*-deficient T_H17 cells compared to control T_H17 cells. Blue bar indicates the –log10 Benjamini-Hochberg–corrected *p*-value. Orange square indicates the ratio of the number of down-regulated genes participating in the indicated pathway to the total

number of genes participating in the same pathway. (B) ConsensusPathDB analysis of human orthologues of the 234 differentially expressed genes. Blue bar and number in parenthesis indicate the –log10 *q*-value and the number of down-regulated genes participating in the indicated pathway, respectively.

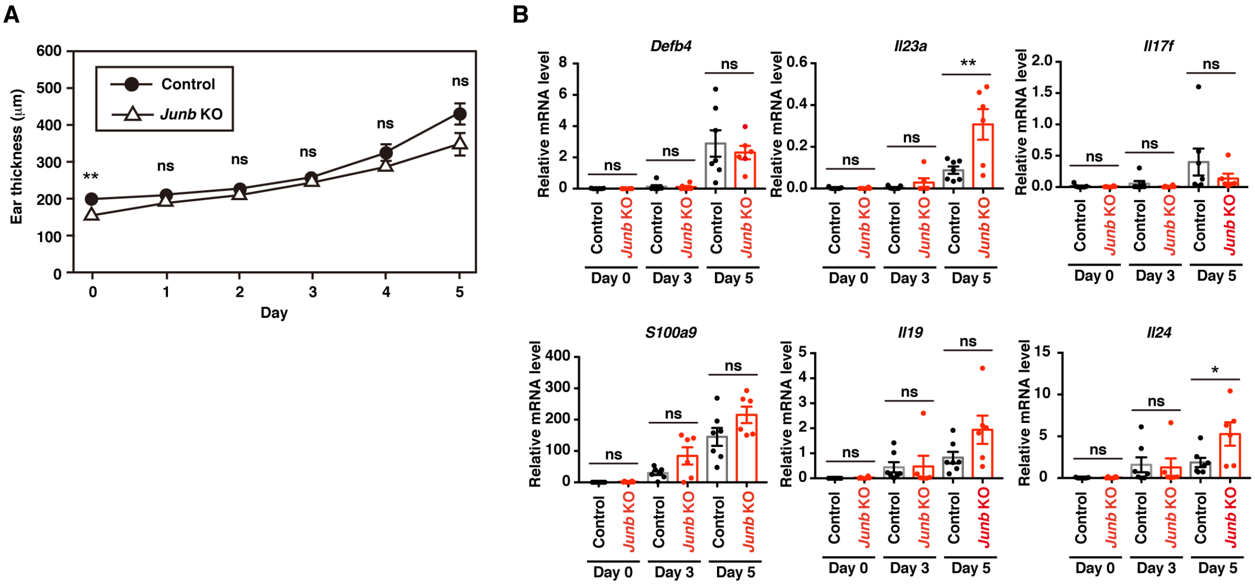

Supplementary Figure 5. Induction of imiquimod-induced psoriasis-like model. (A) Ear thickness of mice treated with imiquimod cream. Data represent the mean of control (n=7) and *Junb*-deficient (n=6) mice. (B) qPCR analysis for expression of the indicated genes in samples obtained by ear biopsy. Expression of *Hprt* was used for normalization. *P<0.05, **P<0.01.

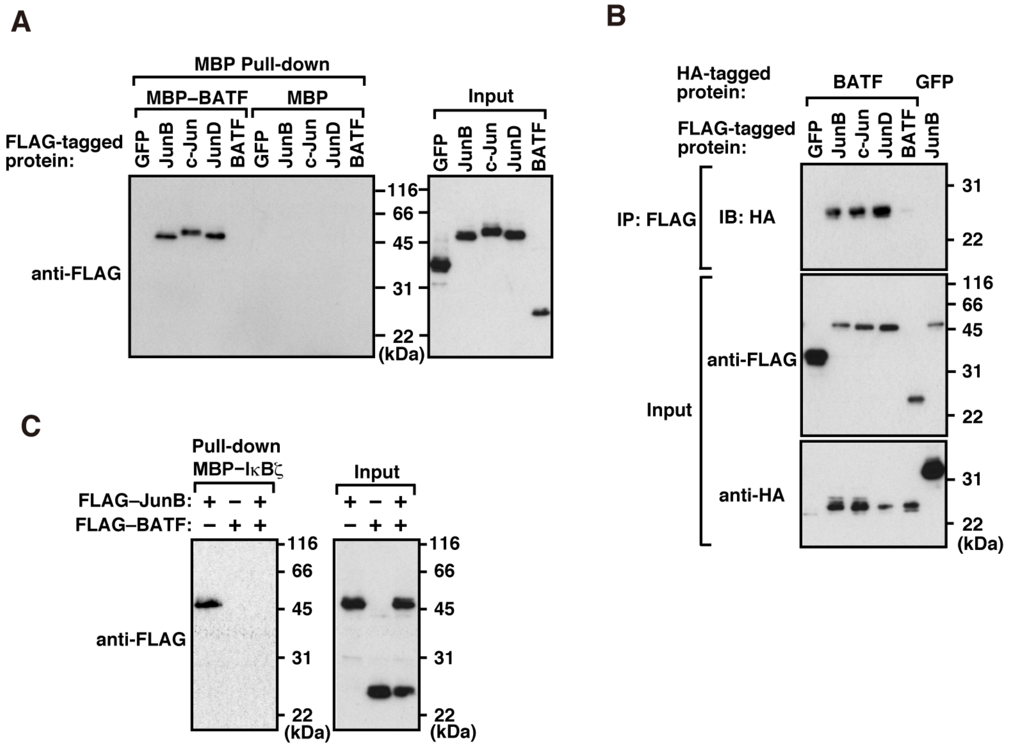

Supplementary Figure 6. Interaction of Jun family proteins with BATF. (A) Bacterially expressed MBP–BATF and *in vitro* translated FLAG-tagged Jun family proteins were mixed and pulled down with Amylose Resin, followed by immunoblot (IB) analysis with anti-FLAG antibody. (B) HEK293T cells were transfected with cDNA for HA–BATF and FLAG-tagged Jun family proteins. Proteins in the cell lysate were subjected to immunoprecipitation (IP) with anti-FLAG antibody, followed by immunoblot (IB) analysis with anti-HA antibody. (C) FLAG–JunB and FLAG–BATF were synthesized separately or simultaneously in an *in vitro* translation system, and mixed with purified MBP–IκBζ. Proteins in the mixture were pulled down with Amylose Resin, followed by immunoblot (IB) analysis with anti-FLAG antibody

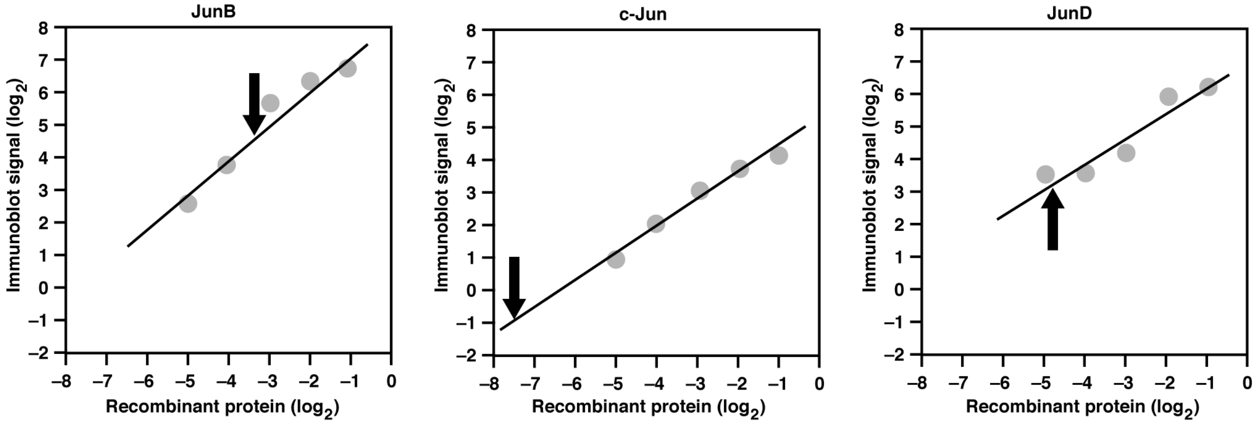

Supplementary Figure 7. Quantification of Jun family proteins expressed in Th17 cells. The signal intensity of an immunoblot band in Figure 5 was quantified using Odyssey Infrared Imaging System (LI-COR), and plotted against the amount of recombinant proteins. The arrows indicate band intensities of endogenous Jun family proteins in the Th17 cell lysate.

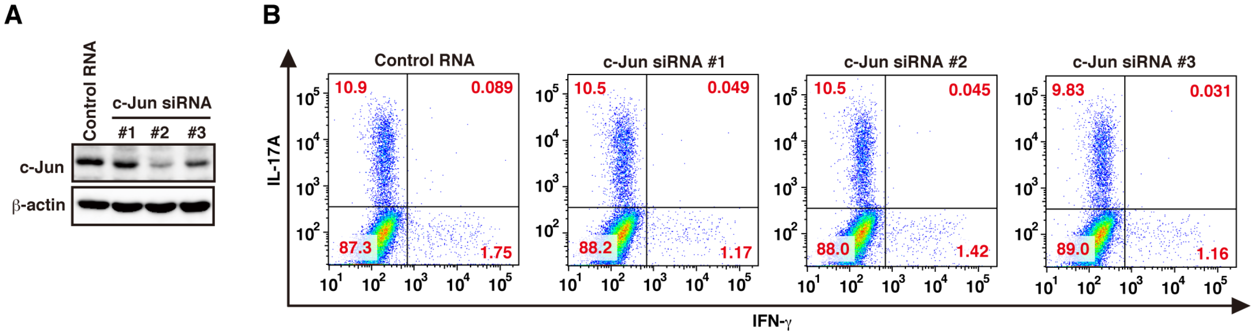

Supplementary Figure 8. Role of c-Jun in Th17 differentiation. (A, B) After transfection with the indicated siRNAs, the $CD4^+$ T cells were cultured under Th17-polarizing conditions. Expression of c-Jun was analyzed by immunobloting (A). Production of IL-17A and IFN-γ was analyzed by intracellular cytokine staining (B).

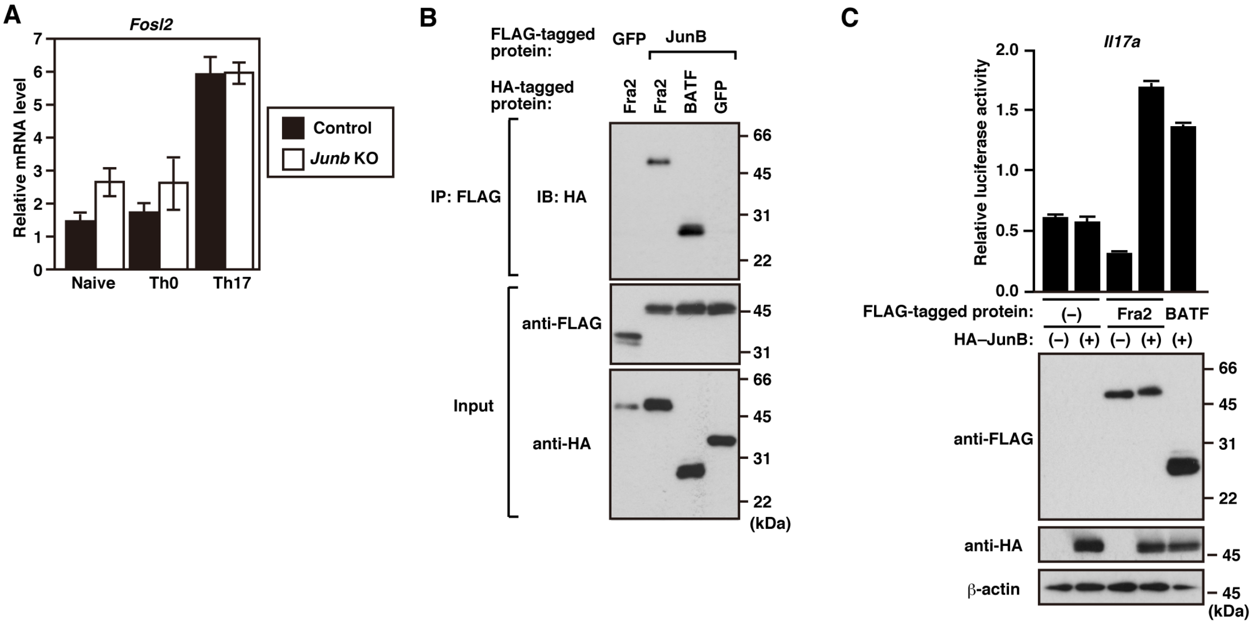

Supplementary Figure 9. Activation of *Il17a* transcription by JunB and Fra2, another AP-1 transcription factor. (A) Expression of *Fosl2* (encoding Fra2) was determined by qPCR. (B) HEK293T cells were transfected with cDNA for HA–BATF and FLAG-tagged Jun family proteins. Proteins in the cell lysate were subjected to immunoprecipitation (IP) with anti-FLAG antibody, followed by immunoblot (IB) analysis with anti-HA antibody. (C) Luciferase reporter assay for transcriptional activation of *Il17a* promoter in HEK293T cells expressing the indicated exogenous proteins and immunoblot (IB) analysis of the exogenously expressed proteins. Data are presented as mean ± SD.

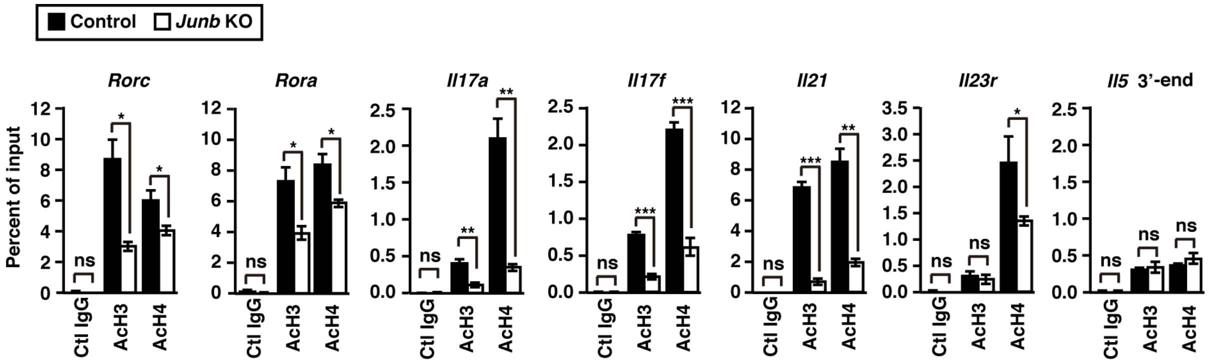

Supplementary Figure 10. Acetylation of histones H3 and H4 at Th17-related gene promoters in *Junb*-deficient CD4$^+$ T cells. Real-time PCR analysis of chromatin immunoprecipitation with anti-acetylated histone H3 or H4 antibody in CD4$^+$ T cells cultured under Th17-polorizing conditions. Data are presented as mean ± SD. *P < 0.05; **P < 0.01; ***P < 0.005; ns, not significant (Student's *t*-test).

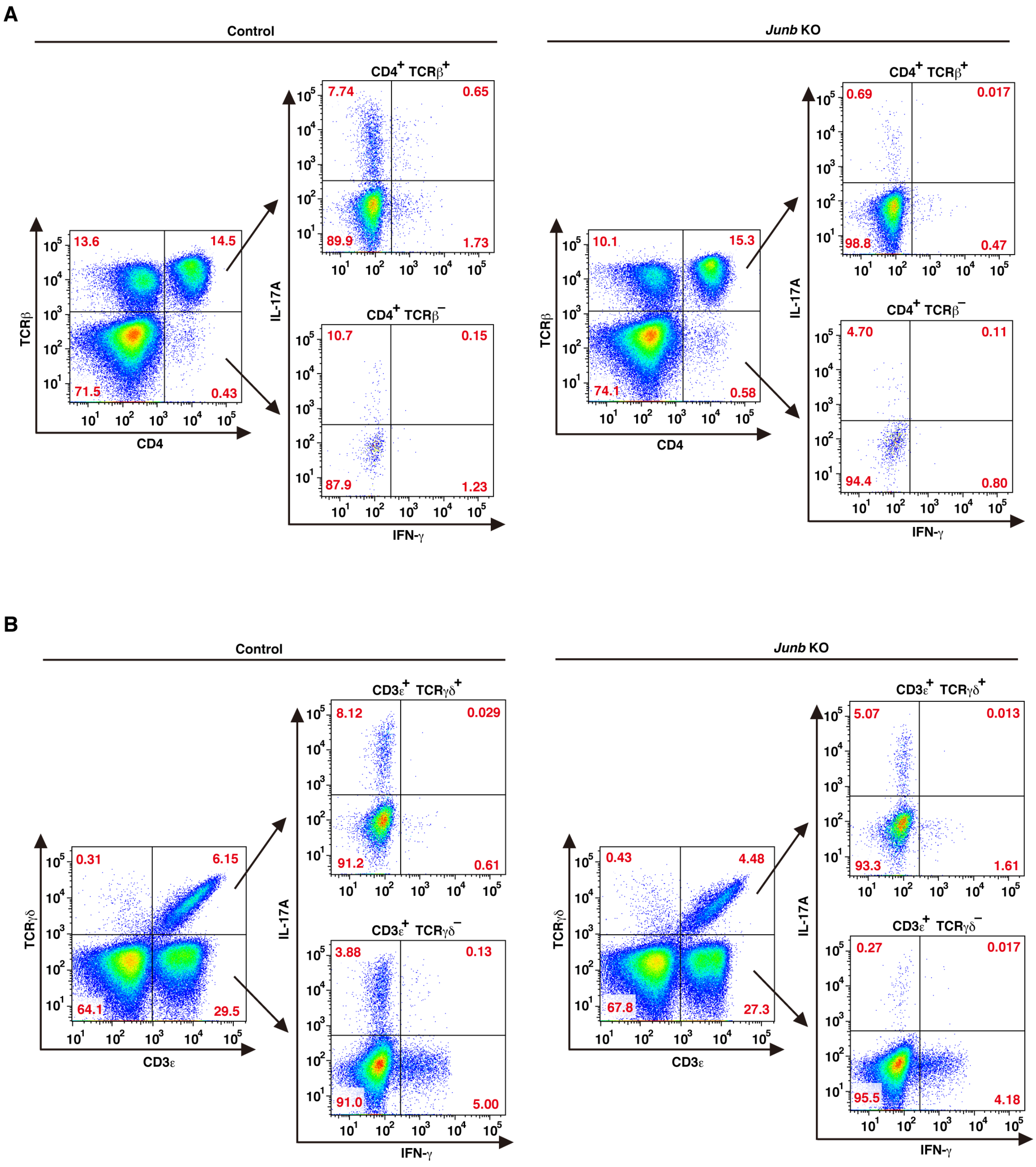

Supplementary Figure 11. IL-17A Production by lymphocytes from short intestinal lamina propria. Lamina propria lymphocytes were prepared from short intestine, and stimulated with PMA and ionomycin in the presence of GolgiPlug. After staining with CD45.2 and the indicated cell surface markers, cells were fixed and analyzed by intracellular cytokine staining. CD45.2^{+} cells were analyzed as indicated.

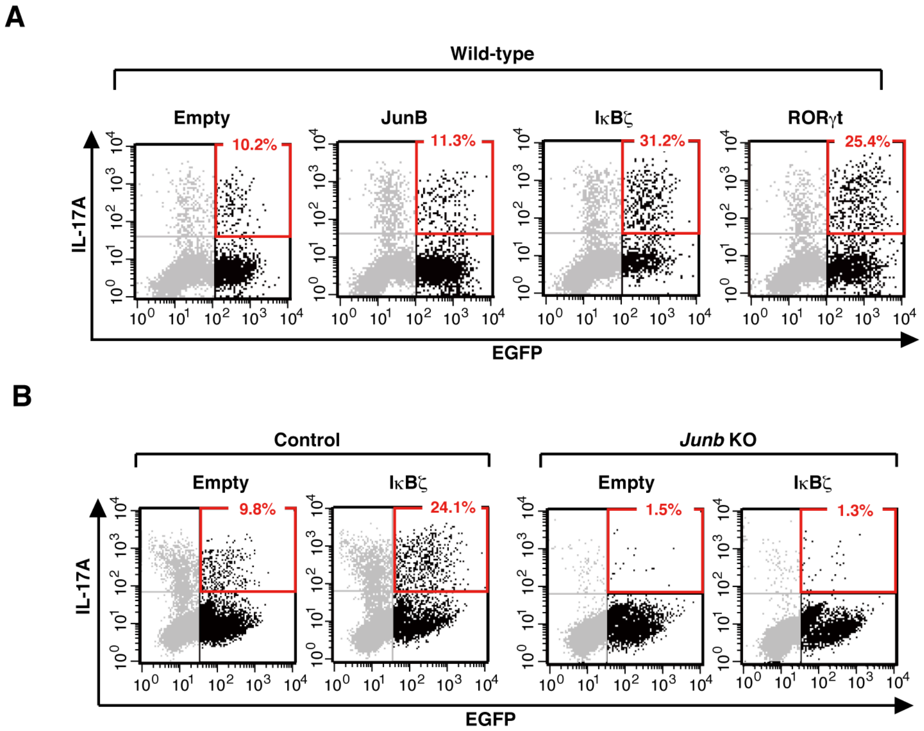

Supplementary Figure 12. Effect of IκBζ overexpression on Th17 cell differentiation. Naive CD4^{+} T cells were prepared from wild-type C57BL/6 mice (A), or *Junb*$^{f/+}$ (control) or *Junb*-deficient (KO) mice (B), and transduced by a bi-cistronic retroviral system for expression of the indicated protein (JunB, IκBζ, or RORγt) together with EGFP under Th17-polarizing conditions. Production of IL-17A was analyzed by flow cytometry; numbers indicate percent EGFP^{+} cells producing IL-17A.
